# Regulatory mechanism of *MeGI* on sexuality in *Diospyros oleifera*

**DOI:** 10.3389/fpls.2023.1046235

**Published:** 2023-02-22

**Authors:** Yini Mai, Peng Sun, Yujing Suo, Huawei Li, Weijuan Han, Songfeng Diao, Liyuan Wang, Jiaying Yuan, Yiru Wang, Lingshuai Ye, Yue Zhang, Fangdong Li, Jianmin Fu

**Affiliations:** ^1^ State Key Laboratory of Tree Genetics and Breeding, Key Laboratory of Non-timber Forest Germplasm Enhancement and Utilization of National Forestry and Grassland Administration, Research Institute of Non-timber Forestry, Chinese Academy of Forestry, Zhengzhou, China; ^2^ Chinese Academy of Sciences (CAS) Engineering Laboratory for Vegetation Ecosystem Restoration on Islands and Coastal Zones, South China Botanical Garden, Chinese Academy of Sciences, Guangzhou, China

**Keywords:** *MeGI*, salicylic acid, circadian rhythm, flavonoids, sex determination

## Abstract

Dioecy system is an important strategy for maintaining genetic diversity. The transcription factor *MeGI*, contributes to dioecy by promoting gynoecium development in *Diospyros lotus* and *D. kaki*. However, the function of *MeGI* in *D. oleifera* has not been identified. In this study, we confirmed that *MeGI*, cloned from *D. oleifera*, repressed the androecium development in *Arabidopsis thaliana*. Subsequently, chromatin immunoprecipitation-sequencing (ChIP-seq), DNA affinity purification-sequencing (DAP-seq), and RNA-seq were used to uncover the gene expression response to *MeGI*. The results showed that the genes upregulated and downregulated in response to *MeGI* were mainly enriched in the circadian rhythm-related and flavonoid biosynthetic pathways, respectively. Additionally, the WRKY DNA-binding protein 28 (*WRKY28*) gene, which was detected by ChIP-seq, DAP-seq, and RNA-seq, was emphasized. *WRKY28* has been reported to inhibit salicylic acid (SA) biosynthesis and was upregulated in MeGI-overexpressing *A. thaliana* flowers, suggesting that *MeGI* represses the SA level by increasing the expression level of *WRKY28*. This was confirmed that SA level was lower in *D. oleifera* female floral buds than male. Overall, our findings indicate that the *MeGI* mediates its sex control function in *D. oleifera* mainly by regulating genes in the circadian rhythm, SA biosynthetic, and flavonoid biosynthetic pathways.

## 1 Introduction

Persimmon (*Diospyros kaki* Thunb.) is one of the most widely distributed fruit tree species in the world. The fruit is sweet and rich in vitamin C and other nutrients; it also has considerable economic value (Han et al., 2016). According to the de-astringency capacity of fruit at the commercially mature stage (fruits turn the color of the mature peel but do not intenerate), persimmon is divided into pollination-constant non-astringent (PCNA) and non-PCNA groups (Akagi et al., 2011). The PCNA group includes Japanese-type PCNA and Chinese-type PCNA subgroups, which are edible without any artificial treatment, and are generally preferred by consumers (Sato and Yamada, 2016). The non-PCNA group is divided into three subgroups according to the effect of the seeds on de-astringency capacity, such as pollination-variant non-astringent, pollination-constant astringent, and pollination-variant astringent (Yonemori et al., 2000).

China has the richest persimmon germplasm resources worldwide, but most cultivars are pollination-constant astringent (Ai et al., 2014). The current development and utilization of persimmon resources in China are extremely insufficient, leading to wasted resources. Thus, there is a need to breed new cultivars with traits such as easy de-astringency, high storability, and good flavor to promote the development of the persimmon industry. However, only 20 persimmon cultivars (approximately 2% of the total) bear male (or hermaphroditic) flowers (Fu et al., 2017), which are valuable pollen donors for cross breeding. This limited availability of the paternal parent has seriously restricted the development of persimmon hybrid and ploidy breeding (Wang et al., 2020a). Thus, the sex differentiating mechanism of persimmon must be explored to support the cultivation of high-quality male resource.

Three main modes of sexual expression exist in persimmons including gynoecious (bearing only female flowers), monoecious (bearing both female and male flowers), and polygamomonoecious (bearing female, male, and hermaphroditic flowers) (Wang et al., 2020a; Sun et al., 2022). We have also determined that a small number of wild persimmon trees (*D. kaki*) are androecious and andromonoecious (i.e., bear staminate flowers only [Wang et al., 2018] and bear both staminate and hermaphroditic flowers [Li et al., 2019], respectively).

The pistil and stamen primordia are produced during the early stages of all persimmon floral buds. The pistil or stamen primordia is aborted during development, leading to the formation of unisexual flowers (Li et al., 2016; Wang et al., 2020b). The *OGI/MeGI* system is the primary switch that determines floral sex. *D. oleifera* and *D. kaki* include dioecious, monoecious, and andromonoecious types (Sun et al., 2022), while *Diospyros lotus* [a closely related species of *D. oleifera* and *D. kaki* (Fu et al., 2016)] contains only dioecious types (Akagi et al., 2014). Therefore, Akagi et al. (2014) established the *OGI/MeGI* regulatory theory with dioecious *D. lotus*; in this theory, the homeodomain transcription factor (TF) *MeGI* (Japanese for ‘female tree’), targeted by small RNAs transcribed by the Y chromosome-specific gene *OGI* (Japanese for ‘male tree’), determines floral sex by a dose effect (Akagi et al., 2014). Moreover, the greatest divergence of *MeGI* expression between female and male floral buds in *D. kaki* is observed in mid-April (Li et al., 2019); this is considered a critical stage for sex differentiation because selective abortion of the pistil or stamen primordia occurs at this time (Li et al., 2016). Accordingly, *MeGI* is important for sex differentiation in persimmon floral buds.

Based on the *OGI/MeGI* theory, Akagi et al. (2016) studied sex differentiation in hexaploid *D. kaki*; they reported that the flower bud sex in hexaploid *D. kaki* is mainly regulated by the level of methylation in the *MeGI* promoter region. Additionally, the methylation level is susceptible to hormones (Ran et al., 2016), water (Wang et al., 2011), temperature (Steward et al., 2002; Hashida et al., 2006; Choi and Sano, 2007), radiation (Aypar et al., 2011), and other external environmental conditions (Aryal and Ming, 2014). Our previous study showed that gibberellin promotes the development of the stamen, whereas zeatin, ethylene, and methylation inhibitors promote pistil development and inhibit stamen development in hexaploid *D. kaki* (Sun et al., 2017; Wang et al., 2020a; Wang et al., 2022). All of these results suggest that flower sex can be artificially regulated in *Diospyros* spp.

Identification of the functions of genes orchestrated by *MeGI* would be helpful for artificial sex control. Yang et al. (2019) used DNA affinity purification-sequencing (DAP-seq) and mRNA-seq to explore the downstream genes regulated by *MeGI*. MADS-box genes, such as *PISTILLATA (PI)* and *AGAMOUS (AG)*, were identified in their study. However, genes enriched in primary and secondary metabolites, plant hormone biosynthesis and signal transduction, and plant-pathogen interaction pathways need further investigation.

The developmental stages of female and male floral buds obtained from the *D. kaki* cultivar (cv.) (‘Zenjimaru’) have been defined (Li et al., 2016). Our previous observations indicated that the development of diploid *D. oleifera* floral buds is synchronous with the development of hexaploid *D. kaki* and also exhibit a similar diversity in gender types as *D. kaki* (Sun et al., 2022). In addition, a new genome of the diploid *D. oleifera* has been assembled at the chromosome scale (Sun et al., 2022). These results suggest that *D. oleifera* is an excellent model for uncovering the mechanisms that underlie *MeGI* function. In this study, we used ChIP-seq and DAP-seq to identify the downstream genes directly regulated by *MeGI*; we also used RNA-seq to determine the gene expression response after *MeGI* overexpression in *Arabidopsis thaliana* flowers. Additionally, we used electrophoretic mobility shift assays (EMSAs) to detect interactions between *MeGI* and the captured downstream genes. Finally, we used the results to explore the mechanisms that underlie *MeGI* function.

## 2 Materials and methods

### 2.1 Plant materials

The second key stage for sexual differentiation [stage 8; initiation of microsporocytes and arrest of carpel primordia indicated by limited size increase (male floral buds); two integuments and macrosporocytes and arrest of outside stamen primordia indicated by limited size increase (female floral buds)] (Li et al., 2016) was observed in female and male floral buds of *D. kaki* in mid-April (April 15-17). The greatest divergence in *MeGI* expression between *D. kaki* female and male floral buds was also observed in mid-April (Li et al., 2019). Thus, female and male floral buds were obtained from gynoecious and androecious *D. oleifera* trees, respectively, at the same developmental stage from the Guangxi Zhuang Autonomous Region, China (**Supplementary Figure S1**) in mid-April. The samples were snap-frozen in liquid nitrogen and stored at −80°C until further analysis. Female buds were used to amplify the *MeGI* coding sequence and conduct the DAP-seq analysis. Female and male buds were used for determination of the salicylic acid (SA) content.

### 2.2 MeGI protein sequence analysis

We extracted RNA from *D. oleifera* flower buds and reverse transcribed it into complementary DNA (cDNA). We cloned the *MeGI* open reading frame (ORF) sequence from the cDNA using the following primers: MeGI-F (5′-ATGACAGCCAACTTTAATCC-3′) and MeGI-R (5′-TCATATAAGGTTAACCCATT-3′). TBtools software (v1.098) (Chen et al., 2020) was used to identify the location of the *D. oleifera* HD-ZIP TF family. The molecular weight and isoelectric point of the MeGI protein sequence were predicted by ExPASy (http://web.expasy.org/protparam/). Multiple sequence alignment and construction of the phylogenetic tree for the MeGI protein with its homologues from other species was performed using MEGA 7.0 software and the neighbor-joining method with 1,000 bootstrap replicates.

### 2.3 Subcellular location analysis

The full-length *MeGI* coding sequence without a stop codon was amplified by polymerase chain reaction (PCR). The primers for PCR amplification were: MeGI-F-pCAM35S (5′-GGGGACGAGCTCGGTACCATGACAGCCAACTTTAATCCTCAG-3′) and MeGI-R-pCAM35S (5′-CATGGTGTCGACTCTAGATATAAGGTTAACCCATTCCATGC-3′). Subsequently, the PCR product was connected to the pCAM35S-green fluorescent protein (GFP) plasmid *via* the KpnI and XbaI restriction sites. The recombinant plasmid was transformed into *Agrobacterium tumefaciens* GV3101 and transiently expressed in tobacco leaf cells using transformed *Agrobacterium* that contained the full-length cDNA of the *D. oleifera* genome. The pCAM35S-GFP empty vector served as the control. Leaf cells were observed and photographed at 48 h using a laser confocal microscope (Olympus, Tokyo, Japan).

### 2.4 Plasmid constructs and genetic transformation

The full-length *MeGI* coding sequence was amplified by PCR and inserted into the pHB vector (**Supplementary Figure S2A**) (driven by the CaMV 35S promoter) using the following primers: MeGI-F-pHB (5′-CGCGGATCCATGACAGCCAACTTTAATCC-3′) and MeGI-R-pHB (5′-CGCGAGCTCTCATATAAGGTTAACCCATTC-3′). It was inserted into the p1306-FLAG vector (**Supplementary Figure S2B**) (driven by the CaMV 35S promoter) using the following primers: MeGI-F-p1306-FLAG (5′-ACGGGGGACGAGCTCGGTACCATGACAGCCAACTTTAATCC-3′) and MeGI-R-p1306-FLAG (5′-TCCAAGGGCGAATTGGTCGACTATAAGGTTAACCCATTCCATG-3′). These two recombinant plasmids were transformed into *A. thaliana* as described by Clough and Bent (1998). Briefly, Columbia-0 ecotype *A.thaliana* plants were grown at 22°C under 150 μmol/M^2^/S illumination intensity and a 16-h light/8-h dark photoperiod. The pHB-MeGI and p1306-FLAG-MeGI constructs were introduced into *A. tumefaciens* GV3101 (Pu Jie Biology, Shanghai, China) using the freeze-thaw method. Then, wild-type *A. thaliana* plants were transformed using the floral-dip method; the pHB and p1306-FLAG empty vectors served as respective controls for the pHB-MeGI and p1306-FLAG-MeGI constructs. Putative transgenic plants were screened on ½ Murashige and Skoog medium containing 50 μg/mL kanamycin. A Stemi 508 stereomicroscope (Zeiss, Oberkochen, Germany) was used to photograph the *A. thaliana* flower buds. *MeGI* expression was detected by reverse transcription quantitative PCR with the following primers: MeGI-PCR-F (5′-GACACCAAGGAGAAGTGGTG-3′) and MeGI-PCR-R (5′-CTCCAGCTTATGTTCGTTCC-3′) for *MeGI*; AtActin2-F (5′-TTCTTCTTACCGAGGCTCCTC-3′) and AtActin2-R (5′-GAATCCAGCACAATACCGGTTG-3′) for *actin*.

### 2.5 Total RNA extraction

Developing flowers (approximately stage 8) of *A. thaliana* (Smyth et al., 1990) were collected from the pHB-MeGI transgenic lines, which had stunted stamens, and the control lines of the T2 generation. The flower buds of the pHB-MeGI transgenic and control lines were divided into three parts as three biological replicates, respectively. Total RNA was purified from plant tissues using the ethanol precipitation method and the CTAB-PBIOZOL reagent in accordance with the manufacturer’s instructions. Total RNA was qualified and quantified using the NanoDrop spectrophotometer and Agilent 2100 Bioanalyser (Thermo Fisher Scientific, Waltham, MA, USA), respectively.

### 2.6 Construction of the long non-coding RNA library

Illumina sequencing libraries were prepared as previously described (Li et al., 2021). In brief, a Ribo-Zero™ Magnetic Kit (Plant Leaf) (Epicentre) was used to treat approximately 1 µg of total RNA per sample to deplete the rRNA. The retrieved RNA was interrupted by adding the First Strand Master Mix (Invitrogen, Carlsbad, CA, USA). First-strand cDNA was generated by reverse transcription with random primers, and second-strand cDNA was synthesized. The synthetic cDNA was subjected to end-repair and was 3’ adenylated. Adapters were attached to the ends of the 3’-adenylated cDNA fragments. Several rounds of PCR amplification were performed using the PCR Primer Cocktail and PCR Master Mix for enrichment of cDNA fragments. Then, the PCR products were purified using Ampure XP Beads. The final library was qualified and quantified. The qualified library was double-sequenced on the BGISEQ platform (Beijing Genomic Institute [BGI], Shenzhen, China).

### 2.7 Transcriptomic data analysis

The sequencing data were filtered using SOAPnuke software (v1.5.2) (Li et al., 2008). Clean reads were mapped to the *Arabidopsis* reference genome (TAIR10.1) using HISAT2 software (v2.0.4) (Kim et al., 2015). Bowtie2 software (v2.2.5) (Langmead and Salzberg, 2012) was used to compare clean reads with BGI-developed genomic databases containing known, novel, coded, and uncoded transcripts. Gene expression levels were calculated by RSEM software (v1.2.12) (Li and Dewey, 2011). DESeq2 software (v1.4.5) (Love et al., 2014) was used for differential expression analysis with thresholds of fold change ≥ 2 and Q ≤ 0.05. Log_2_ (fold change) values were loaded into MAPMAN software (v3.6.0RC1) (Thimm et al., 2004) for metabolic pathway analysis. Gene Ontology (GO) classification and enrichment analyses, Kyoto Encyclopaedia of Genes and Genomes (KEGG) enrichment analyses and key driver analyses (KDAs) of differentially expressed genes (DEGs) were performed using the Dr. Tom system (BGI).

### 2.8 Construction and analysis of the small RNA library

Total RNA was purified, and the small RNA region corresponding to the 18–30 nt band was excised and recovered. Small RNAs of 18–30 nt were sequentially adenylated to 3’ adapters, unique molecular identifiers, and 5’ adapters. It was then transcribed into cDNA for PCR amplification. The PCR products were screened and purified. The final library was qualified and quantified. The final PCR products were sequenced using the DNBSEQ platform (BGI).

The raw sequencing data were filtered: clean tags were mapped to the *Arabidopsis* reference genome (TAIR10.1) and the miRbase databases using Bowtie2 software (Langmead et al., 2009). miRA software (Evers et al., 2015) was employed to predict the novel miRNAs. TAPIR (Bonnet et al., 2010) and TargetFinder (Fahlgren and Carrington, 2010) softwares were used to predict the miRNA target genes. miRNA expression levels were calculated by counting the absolute numbers of molecules using unique molecular identifiers (Kivioja et al., 2012).

### 2.9 Chromatin immunoprecipitation-sequencing (ChIP-seq)

Developing flowers (approximately stage 8) (Smyth et al., 1990) were collected from the p1306-MeGI-FLAG transgenic lines, which had stunted stamens, and the control lines of the T2 generation. The ChIP assays were performed by Wuhan IGENEBOOK Biotechnology Co., Ltd. (Wuhan, China) using a previously described method (Landt et al., 2012). In brief, the 0.5 g samples were washed twice in cold phosphate-buffered saline buffer, cross-linked with 1% formaldehyde at room temperature for 10 min, and quenched with glycine. The samples were lysed, and the chromatin was acquired on ice. Ultrasonographic analysis of the chromatin yielded soluble sheared chromatin (mean DNA length, 200–500 bp). Then, 20 μL of chromatin were stored at –20°C for input DNA, and 200 μL of chromatin were used for immunoprecipitation with anti-FLAG antibodies (F1804, Sigma-Aldrich, St. Louis, MO, USA). Immunoprecipitation reactions were performed overnight at 4°C with 10 μg of antibody. On the next day, 30 μL of protein beads were added, samples were incubated for 3 h, and the beads were washed. Bound material was eluted from the beads in 300 μL of elution buffer, treated with RNase A at 65°C for 6 h, and then treated with proteinase K overnight at 45°C. The sequencing libraries were constructed using immunoprecipitated DNA, in accordance with the protocol supplied with the I NEXTFLEX^®^ ChIP-Seq Library Prep Kit for Illumina^®^ Sequencing (NOVA-5143-02, BioScientific, Austin, TX, USA). Sequencing was performed on the Illumina NovaSeq 6000 using the PE 150 method.

### 2.10 ChIP-seq data analysis

Trimmomatic software (v0.38) was employed to filter out low-quality reads (Bolger et al., 2014). Clean reads were mapped to the *Arabidopsis* reference genome (TAIR10.1) using Bwa software (v0.7.15) (Li and Durbin, 2009). Potential PCR duplicates were removed using Samtools software (v1.3.1) (Li et al., 2009). MACS2 software (v 2.1.1.2) (Zhang et al., 2008) was used to call peaks with the default parameters (bandwidth, 300 bp; model fold, 5, 50; q-value, 0.05). If the summit of a peak was closest to the transcription start site of one gene, the peak was assigned to that gene (Salmon-Divon et al., 2010). DiffBind software (v1.16.3) (Stark and Brown, 2011) was used to analyze the peaks of two different groups of samples. HOMER (v3) was employed to predict the occurrence of a motif within peaks, using the default settings for a maximum motif length of 12 base pairs (Hull et al., 2013). GO enrichment analysis was performed using the EasyGO gene ontology enrichment analysis tool (http://bioinformatics.cau.edu.cn/easygo/) (Zhou and Su, 2007). The ClusterProfiler package (http://www.bioconductor.org/packages/release/bioc/html/clusterProfiler.html) in R software (Yu et al., 2012) was used for KEGG (http://www.genome.jp/kegg/) enrichment analysis (Altermann and Klaenhammer, 2005).

### 2.11 DNA affinity purification-sequencing

The DAP genomic DNA library was prepared by Zoonbio Biotechnology Co., Ltd. (Nanjing, China), and the DAP reaction was completed as previously described with minor modifications (O’Malley et al., 2016). The optimized version of the BioNano optical mapping-assisted assembly of the genome was used as the reference genome (Sun et al., 2022). Briefly, the Diagenode Bioruptor UCD-300 (Plus) was used to fragment the gDNA to 100–400 bp. The setting was up 10 cycles; each cycle included 30 s of ultrasonic time and a 30-s time interval. End-repair was performed and the dA-tail of the resulting fragmented gDNA was added using the NEXTflex Rapid DNA-Seq Kit (BioScientific). Then, the DAP-seq adaptor was ligated to the fragmented gDNA with the NEXTflex ligase Enzyme Mix (BioScientific). Full-length *MeGI* cDNA was cloned and transferred to pDAP-Halo-Kan to generate pDAP-Halo-Kan-MeGI. The N-terminal Halo-tagged *MeGI* was produced using the TNT SP6 Wheat Germ Master Mix (Promega, Fitchburg, WI, USA) after incubation for 2 h at 25°C. Halo-MeGI was immobilized on magnetic anti-Halo-Tag beads, then washed and incubated with the DNA library. After the beads had been washed, the DNA was eluted and amplified with the indexed TruSeq primers. Sequencing was performed on the Illumina NovaSeq 6000 using the PE 150 method. This experiment was repeated twice (MeGI-1 and MeGI-2). GO classification analysis was performed using TBtools software (v1.098) (Chen et al., 2020).

### 2.12 Electrophoretic mobility shift assay

EMSAs were performed as previously described (An et al., 2018). The *MeGI* cDNA was introduced into *p*CZN1, and the recombinant His-MeGI was purified with Ni-NTA HIS*BIND RESIN (Novagen, Madison, WI, USA), in accordance with the manufacturer’s instructions. EMSAs were performed using the LightShift^®^ Chemiluminescent EMSA Kit (Thermo Scientific). The sequences of the biotin-labelled probes (wild-type: probe-w and mutant: probe-m) (Zoonbio Biotechnology, Nanjing, China) are shown in **Supplementary Table S1**. Unlabelled competitors (wild-type and mutant) were added at 100-fold excess concentrations.

### 2.13 Measurement of salicylic acid content

The SA contents of female and male *D. oleifera* flowers buds were determined by double-antibody sandwich enzyme-linked immuno sorbent assays based on the instructions with the Plant Salicylic Acid ELISA Kit (YS04063B; YaJi Biological, Shanghai, China). Three biological replicates were performed for each sample.

## 3 Results

### 3.1 MeGI sequence analysis

**Figure 1:**
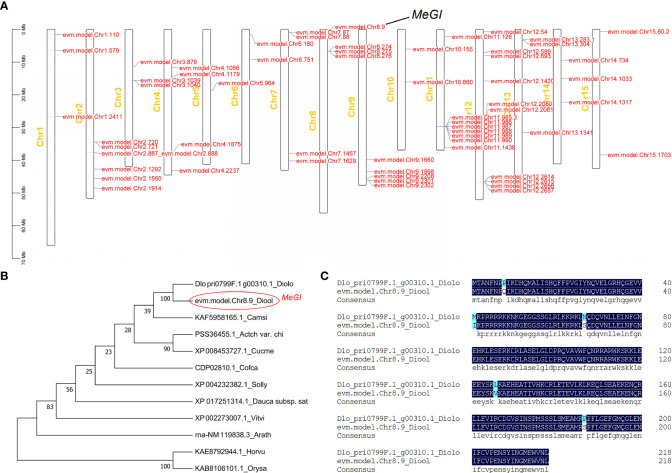
Location of the HD-ZIP transcription factor family and phylogenetic analyses of the MeGI protein. **(A)** Location of the HD-ZIP transcription factor family. **(B)** Phylogenetic analysis of the MeGI protein with its optimal homologues from other species. Diolo, *Diospyros lotus*; Diool*, Diospyros oleifera;* Camsi, *Camellia sinensis*; Actch var. Chi, *Actinidia chinensis* var. *Chinensis*; Cucme, *Cucumis melo*; Cofca, *Coffea canephora*; Solly, *Solanum lycopersicum*; Dauca subsp. Sat, *Daucus carota subsp. Sativus*; Vitvi, *Vitis vinifera*; Arath, *Arabidopsis thaliana*; Horvu, *Hordeum vulgare*, and Orysa, *Oryza sativa.* **(C)** Sequence alignment analysis of the MeGI protein from *D*. *oleifera* and *D*. *lotus.* Diolo*, Diospyros lotus;* Diool*, Diospyros oleifera.*.

In this study, we cloned the *MeGI* ORF sequence from the cDNA of *D. oleifera* female flowers; the sequence is shown in **Supplementary Figure S3**. Yang et al. (2019) reported that *MeGI* belongs to the HD-ZIP TF family. We analyzed the location of the HD-ZIP TF family in *D. oleifera*; we detected 60 HD-ZIP TFs distributed on 15 chromosomes, among which *MeGI* was distributed on chromosome 8 (**Figure 1A**). The *MeGI* ORF was 660 bp in length and encoded 220 amino acids. The molecular weight of the MeGI protein was 25.34 kDa, and the isoelectric point was 9.22. A phylogenetic tree was constructed to assess the evolutionary relationships of MeGI protein with its optimal homologues from *Diospyros lotus*, *A. thaliana*, *Solanum lycopersicum*, *Coffea canephora*, *Daucus carota subsp. Sativus*, *Actinidia chinensis* var. *Chinensis*, *Camellia sinensis*, *Vitis vinifera*, *Cucumis melo*, *Hordeum vulgare*, and *Oryza sativa* (Suo et al., 2020). Phylogenetic analysis showed that the *D. oleifera* MeGI protein had the closest evolutionary relationship with Dlo_pri0799F.1_g00310.1, which is the *D. lotus* MeGI protein, and the most distant evolutionary relationship with KAE8792944.1 of *Hordeum vulgare* and KAB8106101.1 of *Oryza sativa* (**Figure 1B**). We compared the MeGI protein sequences of *D. oleifera* and *D. lotus*; the alignment rate was 97.72% (**Figure 1C**).

### 3.2 Subcellular localisation of the MeGI protein

**Figure 2:**
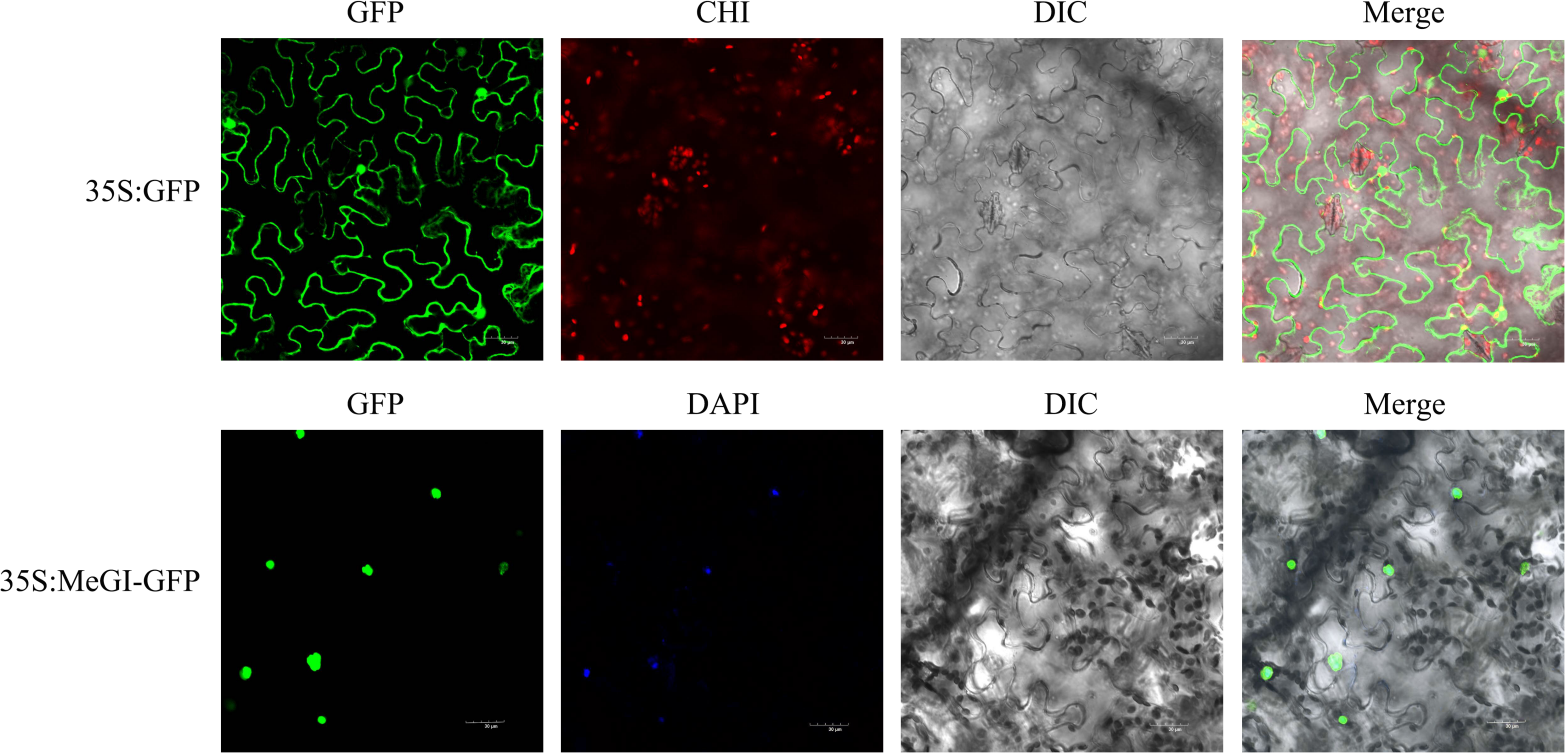
Subcellular localisation of the MeGI protein in tobacco mesophyll cells. The fusion protein (MeGI-GFP) was transiently expressed in tobacco. DAPI was used as the nuclear localisation marker. GFP, Green fluorescence field; DAPI, DAPI field (nuclear staining); CHI, Chloroplast autofluorescence field; DIC, Bright field; Merge, superposition field. Bars, 30 μm.

The subcellular localisation of the *D. oleifera* MeGI protein was analyzed by transient expression of the GFP fusion protein (MeGI-GFP) in tobacco leaf epidermal cells. Confocal laser microscopy observations showed that the MeGI-GFP fusion protein fluoresced in the tobacco nucleus, indicating that the MeGI protein was located in the nucleus and co-localised with the nuclear stain 4′,6-diamidino-2-phenylindole (DAPI) (**Figure 2**).

### 3.3 Gene activation by ectopic expression of *MeGI* in *Arabidopsis*

**Figure 3:**
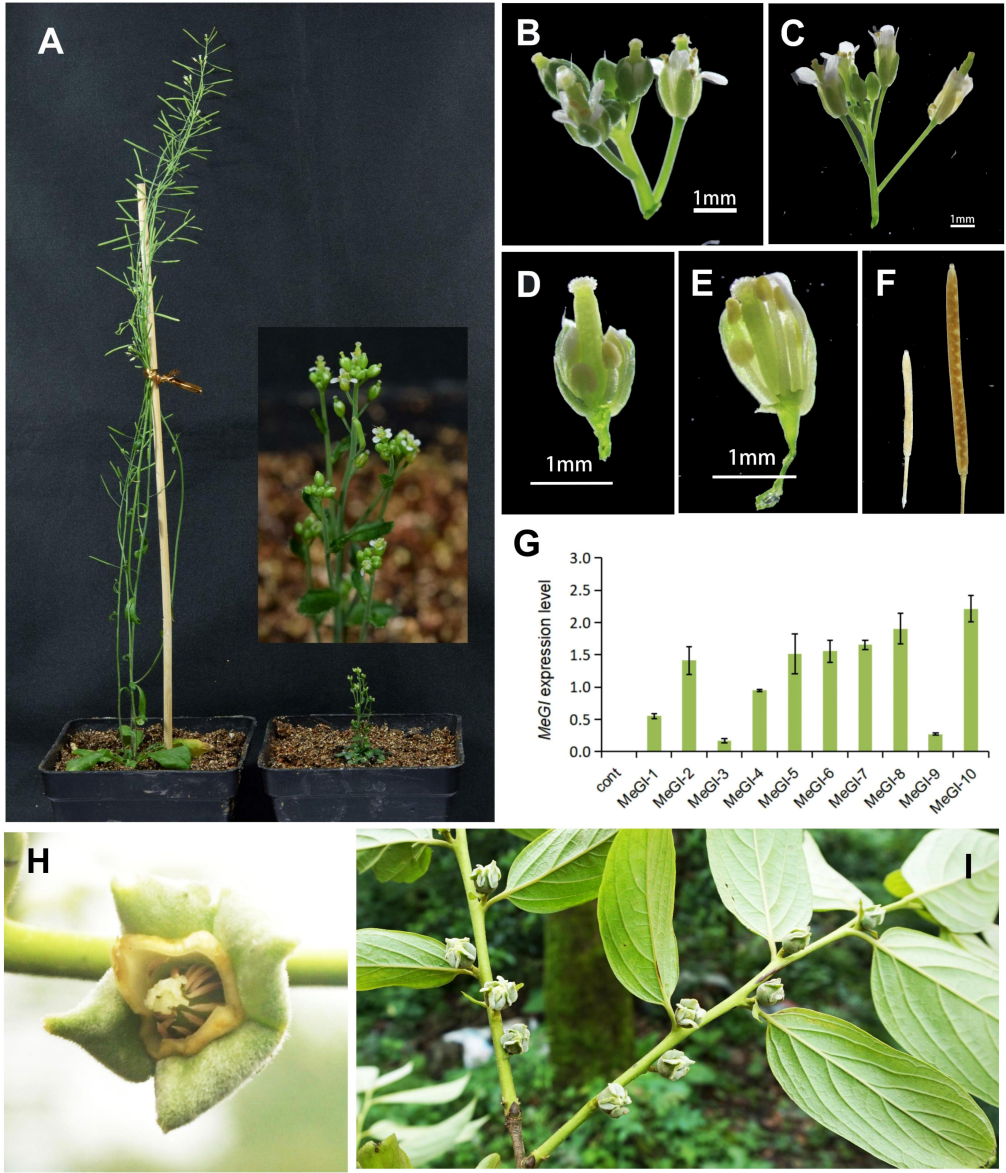
Phenotypes of *A. thaliana* plants overexpressing *MeGI* driven by the CaMV 35S promoter. **(A)** Whole plants. Left, hermaphrodite control plant; right, feminized plant (35S-MeGI-10). **(B, C)** Flowers with defectively developed stamens and petals **(B)** and control flowers **(C)**. **(D, E)** Dissected flowers of the MeGI-OX **(D)** and control **(E)** lines. **(F)** Siliques and seeds. Left, stunted silique of the MeGI-OX line; right, control line. **(G)** *MeGI* expression levels in 35S-empty control and 35S-MeGI-OX lines. Bars indicate standard errors. **(H, I)** The morphology of *D*. *oleifera* female flowers.

Previous studies have shown that the overexpression of *MeGI* from diploid *D. lotus*, driven by the CaMV 35S promoter, suppresses the development of anthers and petals in *A.thaliana* (Akagi et al., 2014; Yang et al., 2019). To identify the function of *MeGI* obtained from diploid *D. oleifera*, we cloned the full-length coding sequence of *MeGI* from *D. oleifera* and transfected it into *A.thaliana.* The results showed that 3 of 10 A*. thaliana* plants transfected with *MeGI* driven by the CaMV 35S promoter were stunted during stamen development (**Supplementary Table S2**), and one was severely dwarfed (35S-MeGI-10) (**Figures 3A, B, D**). However, these plants produced some viable pollen grains, and the carpels produced a few fertile seeds when self-pollinated (**Figure 3F**). The other 7 A*. thaliana* transformants carrying the same overexpressed structure and the control lines grew normally (**Figures 3A, C, E**); they also had typical male fertility (**Figure 3F**). *MeGI* expression levels in 35S-empty control and 35S-MeGI-OX lines were shown in **Figure 3G**. In conclusion, the phenotypes of transgenic *A.thaliana* agreed with the morphology of the *D. oleifera* female flowers (**Figures 3H, I**).

### 3.4 RNA-seq-mediated identification of differentially expressed mRNAs, lncRNAs and miRNAs

**Figure 4:**
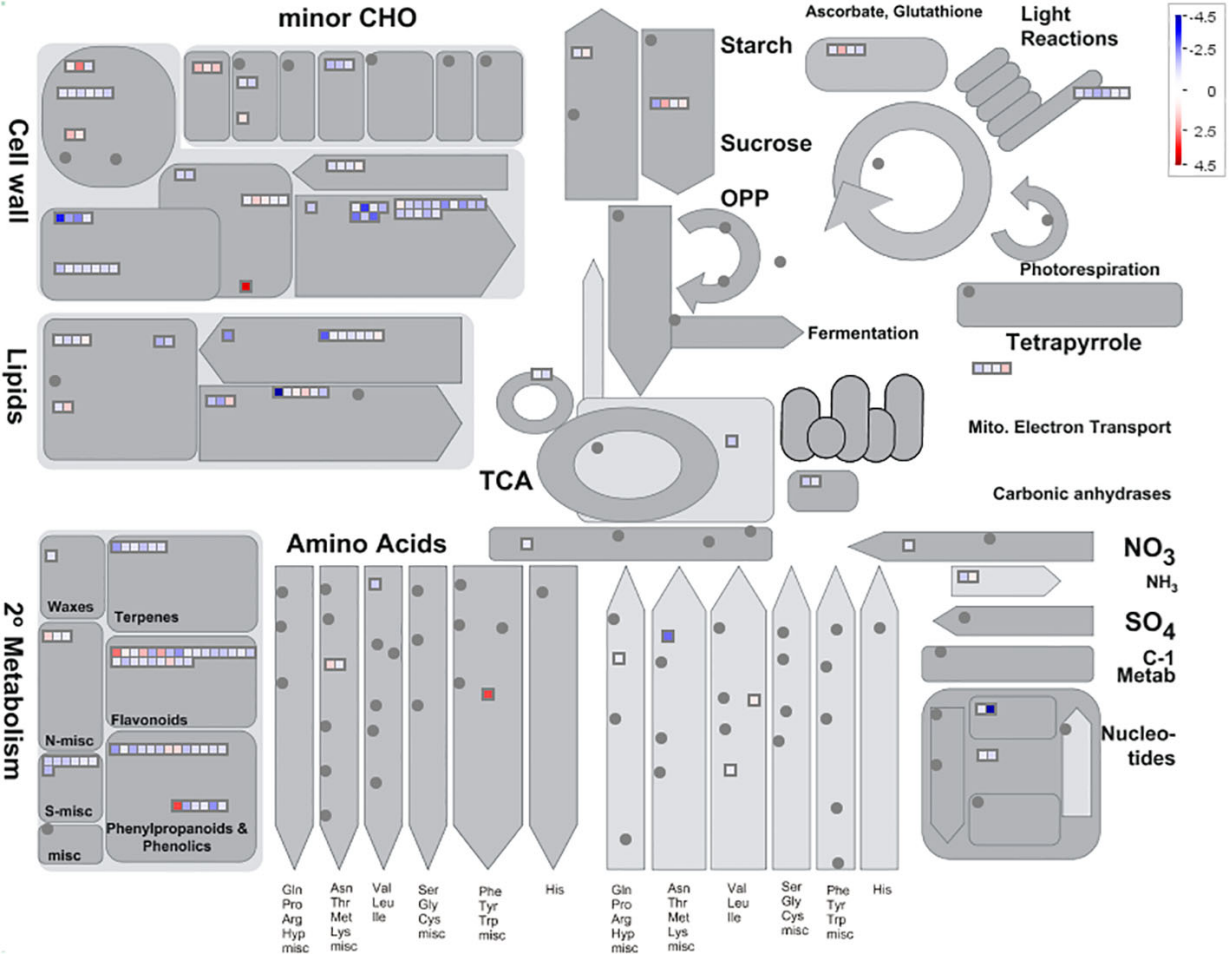
Metabolic pathways with different expression profiles in the pHB-MeGI and pHB-empty lines (blue, pHB-empty biased; red, pHB-MeGI biased). Each square represents a differentially expressed gene (DEG). See Usadel et al. (2009) for more information concerning interpretation of the MAPMAN diagram.

To investigate the downstream target genes regulated by *MeGI*, we performed whole transcriptome analysis of the pHB-MeGI and pHB-empty control lines using developing *A.thaliana* flower buds collected at approximately stage 8 (Smyth et al., 1990; Yang et al., 2019); each data set was procured from three biological replicates. After sequencing and alignment with the *A.thaliana* genome (TAIR10.1), low-quality reads were eliminated; in total, 113.11, 113.10, 113.49, 117.52, 117.53, and 113.26 Mb of clean reads were obtained (**Supplementary Table S3**). Using the thresholds of |log2 (MeGI_OX/Cont)| > 1 and q-value < 0.05, 1,128 DEGs were identified; among them, 734 and 394 genes were inhibited and induced by *MeGI*, respectively (**Supplementary Figures S4A, B**). MapMan analysis showed that the DEGs were mainly concentrated in the cell wall, lipid metabolic, and secondary metabolic pathways (**Figure 4**); the greatest concentration of DEGs was detected in the flavonoid metabolic pathway (**Supplementary Figure S5**). Moreover, the DEGs were classified according to GO analysis (**Supplementary Figure S4C**). In the biological process category, the DEGs were highly clustered in metabolic processes, cellular processes, responses to stimulus, and biological regulation. In the cellular components category, the DEGs were highly clustered in cells and cell parts. In the molecular functions category, the DEGs were highly clustered in catalytic activity and binding. Additionally, KEGG enrichment analysis of the DEGs (**Supplementary Figure S4D**) showed significant enrichment (q < 0.05) in circadian rhythms, secondary metabolite biosynthesis, and phenylpropanoid and flavonoid biosynthesis.

Furthermore, we identified 8 differentially expressed lncRNAs according to the standards describe above (**Supplementary Table S4**); we also identified 29 differentially expressed miRNAs (**Supplementary Table S5**). Prediction of miRNA target genes revealed that 7 miRNAs and their target genes exhibited concurrent differential expression (**Supplementary Table S6**).

### 3.5 ChIP-Seq-mediated identification of *MeGI* genomic binding sites

To identify the genome-wide *MeGI*-binding sites, we used transgenic lines expressing the MeGI-FLAG fusion gene under control of the CaMV 35S promoter to perform ChIP-seq. Analysis of flower bud development in the transgenic plants showed that the MeGI-FLAG fusion protein had full *MeGI* activity, similar to the pHB-MeGI line phenotype (**Supplementary Table S7**). The inflorescences of the p1306-MeGI-FLAG and p1306-FLAG lines were collected at stage 8 (Smyth et al., 1990; Yang et al., 2019). The IP_MeGI_FLAG library had 39.7 million reads and the IP_1306_FLAG library had 37.4 million reads; > 93% of the reads were mapped to the *Arabidopsis* genome (TAIR10.1). Additionally, IP_1306_FLAG DNA was used as the negative control to remove the non-specific peaks; 9,769 unique peaks that could potentially bind to *MeGI* were identified. The distribution of these peaks was visualized using the Circos tool (Krzywinski et al., 2009) (**Supplementary Figure S6A**). The genomic locations of peak sequences in the *Arabidopsis* genome were investigated, along with the corresponding functional elements. Overall, 60.63% of the peaks were distributed in promoter regions, and 16.09% were in exon regions; and remaining peaks were in the intergenic, 5′ untranslated region, 3′ untranslated region, intron, and coding sequence regions (**Supplementary Figure S6B**). Moreover, 7,590 genes were closely associated with these ChIP-seq peaks. The results of TF prediction of these genes indicated that these genes mainly belonged to the AP2/ERF, bHLH, MYB, and WRKY TF families (**Supplementary Figure S6C**). In total, 1,630 of 7,604 genes were annotated in the KEGG analysis. According to KEGG pathway analysis of these genes, the plant hormone signal transduction, circadian rhythm, and MAPK signaling pathways were highly enriched (**Supplementary Figure S6D**). Additionally, 118 specific binding motifs enriched in the *MeGI*-binding region were discovered; motifs with p-values in the top 10 are shown in **Supplementary Figure S6E**.

### 3.6 DAP-seq-mediated identification of MeGI genomic binding sites

**Figure 5:**
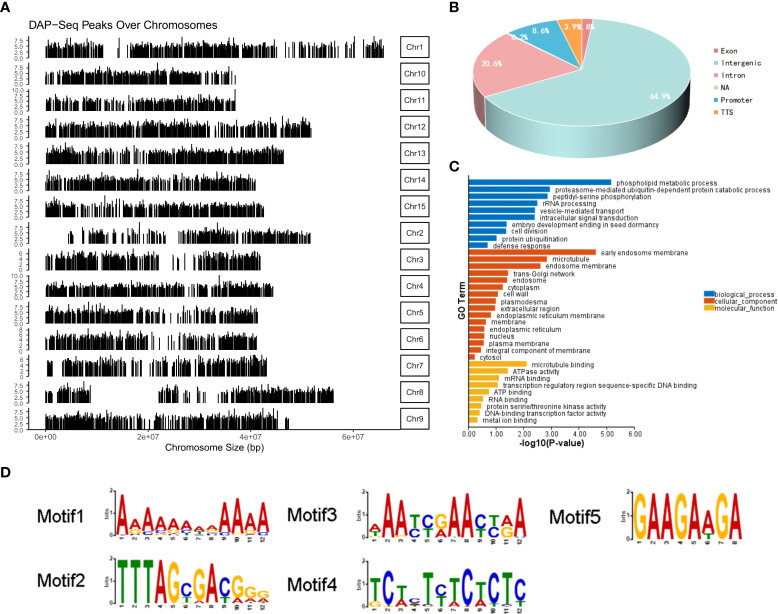
DAP-seq analysis of *MeGI*. **(A)** Distribution of the *MeGI*-binding sites along the *D*. *oleifera* chromosomes. **(B)** Distribution of the *MeGI*-binding sites in the genic and intergenic regions. **(C)** GO classification analysis of the *MeGI*-regulated target genes. **(D)** Nucleotide motifs recognized by *MeGI* in DAP-seq analysis.

*MeGI* in combination with a Halo-Tag was used for the DNA DAP-seq analysis (D’Inca et al., 2021) to examine whether *MeGI* directly targeted any genes. MACS2 was used to analyze the *MeGI*-binding sites. Over the entire *D. oleifera* genome, 7,531 peaks were identified from two repeated experiments, MeGI-1 and MeGI-2 (**Figure 5A**); only 846 peaks were present in both MeGI-1 and MeGI-2. Of the 846 peaks identified, 63 (7.54%) were within the promoter region (2 kb upstream from the transcription start site) (**Figure 5B**). Additionally, 5 specific binding motifs enriched in the *MeGI*-binding regions were discovered (**Figure 5D**). We screened the peaks with MeGI-1 and MeGI-2, as well as a motif matching score (the matching degree between motif and target position, and for the same motif, the better the matching is, the larger the score is) > 10 within the promoter region (2 kb upstream from the transcription start site); we identified the genes nearest to these peaks. Eventually, 58 genes were identified, indicating that *MeGI* binds directly to the promoters of these genes to regulate their expression patterns. The expression levels of these genes in female gynoecious flowers and male androecious flowers (unpublished) were used for reference (**Supplementary Table S8**), and among the 58 genes, using the thresholds of |log2 (Female/Male)| > 1 and q-value < 0.05, seven genes (evm.model.Chr9.1233, evm.model.Chr2.622, evm.model.Chr11.1131, evm.model.Chr2.437, evm.model.Chr1.187, evm.model.Chr13.143, evm.model.Chr4.1147) were up-regulated and 1 gene (evm.model.Chr14.835) was down-regulated. Furthermore, only 3 of the 58 genes were predicted to be transcription factors. Among them, evm.model.Chr5.1036 belonged to the WRKY family, evm.model.Chr7.423 belonged to the bZIP family, and evm.model.Chr14.1204 belonged to the AP2/ERF family. Further GO classification analysis revealed that these 58 genes could be classified into 10 biological process terms, 16 cellular components terms, and 9 biological process terms (**Figure 5C**).

### 3.7 Identification of direct MeGI targets by cross-referencing the ChIP-seq, RNA-seq and DAP-seq data

**Figure 6:**
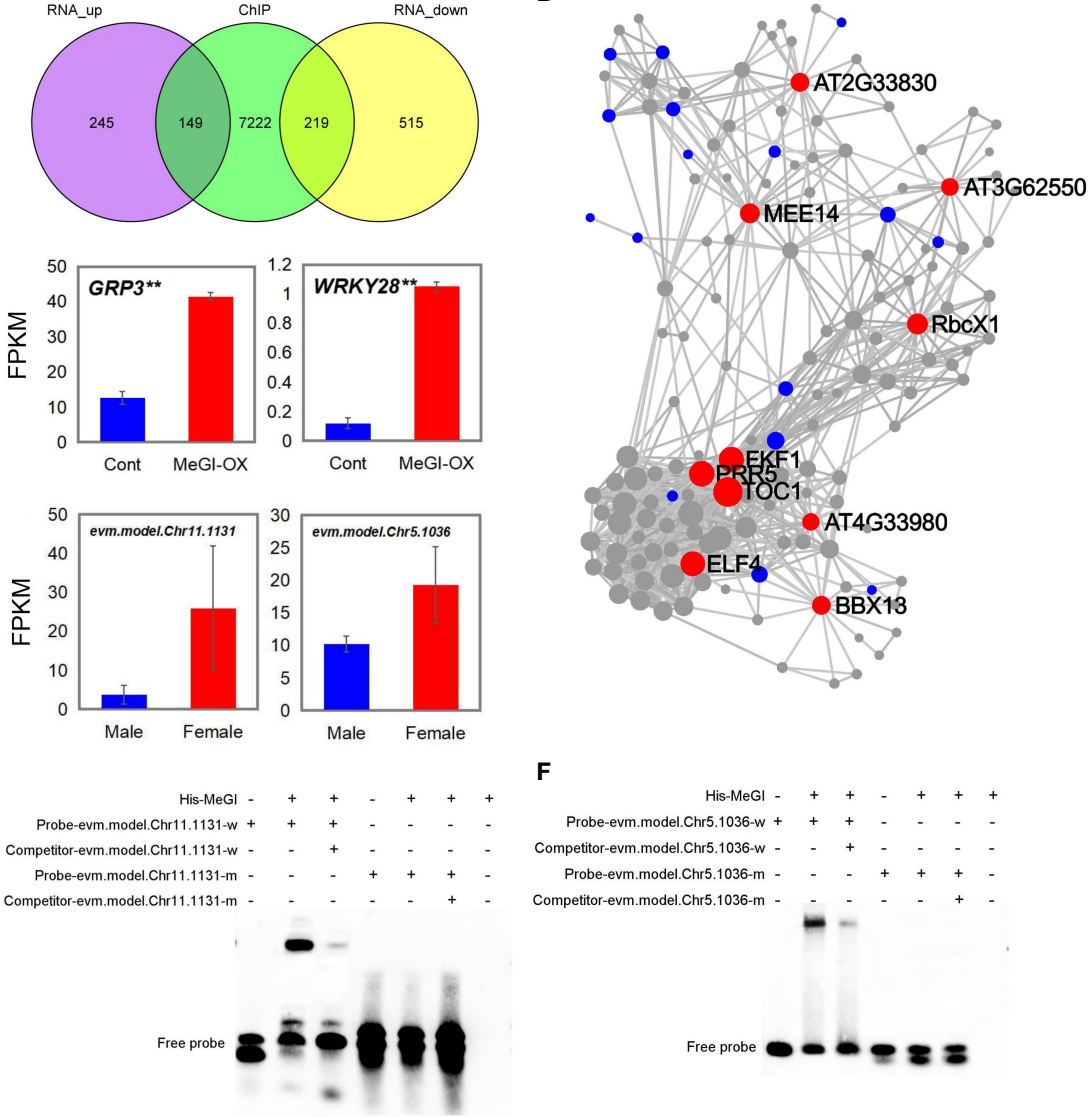
Genes shared among the ChIP-seq, RNA-seq and DAP-seq results. **(A)** Genes shared between ChIP-seq and RNA-seq analyses. **(B)** KDA of 149 upregulated genes; red, blue, and gray circles represent the key driver, initial, and associated genes, respectively. **(C, D)** Fragments per kilobase of transcript per million mapped reads (FPKM) of the shared genes in the ChIP-seq, RNA-seq, and DAP-seq data in MeGI-OX lines and control lines **(C)**, and in female and male flower buds of *D*. *oleifera***(D)**. Bars indicate standard errors. **(E, F)** EMSA results confirming that *MeGI* directly bound to the motifs of the evm.model.Chr11.1131 (*GRP3*) **(E)** and evm.model.Chr5.1036 (*WRKY28*) **(F)** promoters.

Genes that were concurrently identified by ChIP-seq, RNA-seq and DAP-seq are predicted to be targets of *MeGI* regulation. Here, 368 genes were shared between the DEGs from RNA-seq and the bound genes from ChIP-seq. Among the 368 genes, 149 were upregulated and 219 were downregulated in the RNA-seq data (**Figure 6A**; **Supplementary Tables S9**, **10**).

KEGG and GO biological process enrichment analyses and a KDA were performed for the 149 upregulated and 219 downregulated genes, respectively. KEGG enrichment analysis showed that the 149 upregulated genes were significantly enriched in the circadian rhythm and MAPK signaling pathways (**Supplementary Figure S7A**); the 219 downregulated genes were significantly enriched in flavonoid biosynthetic, circadian rhythm, alpha-linolenic acid metabolic, and carotenoid biosynthetic pathways (**Supplementary Figure S7B**). The circadian rhythm pathway was enriched in both upregulated and downregulated genes. GO biological process enrichment analysis showed that the 149 upregulated genes were significantly enriched in rhythmic processes (GO: 0048511), circadian rhythms (GO: 0007623), response to oxidative stress (GO: 0006979), and response to SA (GO: 0009751) (**Supplementary Figure S7C**); the 219 downregulated genes were significantly enriched in response to chitin (GO: 0010200), circadian rhythms (GO: 0007623), and response to auxin (GO: 0009733) (**Supplementary Figure S7D**). The circadian rhythm pathway was enriched in both upregulated and downregulated genes. The KDA results showed that genes such as TIMING OF CAB EXPRESSION 1 (*TOC1*), flavin-binding, kelch repeat, f box 1 (*FKF1*), pseudo-response regulator 5 (*PRR5*), EARLY FLOWERING 4 (*ELF4*), B-box domain protein 13 (*BBX13*), and maternal effect embryo arrest 14 were the key driver genes of the 149 upregulated genes (**Figure 6B**). Among these genes, *TOC1*, *FKF1*, *PRR5*, *ELF4*, and *BBX13* were related to the circadian rhythm pathway. Genes such as 1-aminocyclopropane-1-carboxylic acid synthase 6 (*ACS6*), WRKY DNA-binding protein 40 (*WRKY40*), and cytochrome BC1 synthesis1 (*BCS1*) were the key drivers of the 219 downregulated genes (**Supplementary Figure S8**). Taken together, these results suggest that *MeGI* regulates flower bud differentiation by regulating the expression of circadian rhythm-related genes (e.g., *TOC1*, *FKF1*, *PRR5*, *ELF4*, and *BBX13*).

Next, we annotated the genes identified by DAP-seq by comparison with the *Arabidopsis* genome to identify homologous genes (**Supplementary Table S8**); we compared the homologous genes with the genes shared between ChIP-seq and RNA-seq results. Glycine-rich protein 3 (*GRP3*, evm.model.Chr11.1131) and WRKY DNA-binding protein 28 (*WRKY28*, evm.model.Chr5.1036) were concurrently identified in the ChIP-seq, RNA-seq, and DAP-seq results; both showed higher expression levels in the flowers of the MeGI-OX lines, compared with the control lines (**Figure 6C**). The expression levels of evm.model.Chr11.1131 and evm.model.Chr5.1036 in female and male flower buds of *D. oleifera* were shown in **Figure 6D**. Additionally, the expression levels of evm.model.Chr11.1131 and evm.model.Chr5.1036 were higher in female *D. oleifera* flower buds than in male buds (**Supplementary Table S8**), which was consistent with the RNA-seq results in *A. thaliana*. The GO functional annotation of *GRP3* and *WRKY28* is shown in **Supplementary Table S11**. The promoter regions of evm.model.Chr11.1131 and evm.model.Chr5.1036 were confirmed to be bound by *MeGI via* EMSAs (**Figures 6E, F**), indicating that these genes could be directly regulated by *MeGI.*

### 3.8 SA synthesis analysis and measurement of SA content

**Figure 7:**
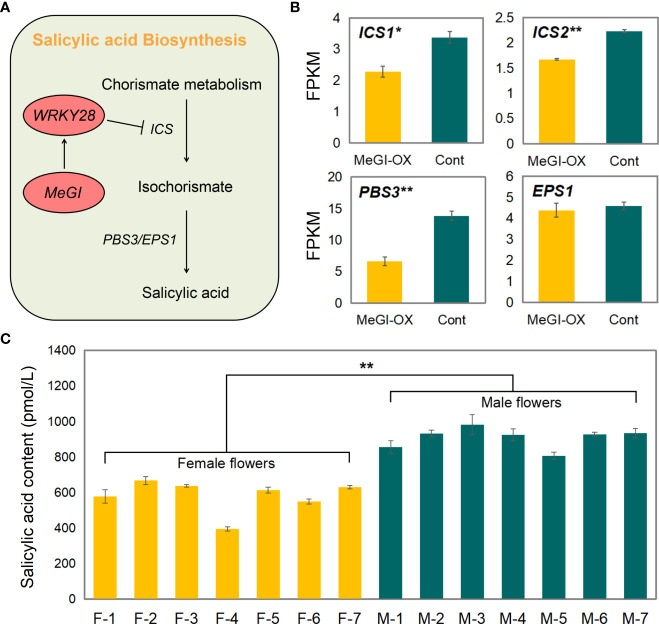
SA biosynthetic pathway and SA content. **(A)** Representative genes in the SA biosynthetic pathway and the model for regulation of SA biosynthesis by *WRKY28*. **(B)** Expression levels of *ICS1, ICS2*, *PBS3*, and *EPS1* in the MeGI-OX and control lines. Bars indicate standard errors. **(C)** SA content in female and male *D*. *oleifera* flowers. Bars indicate standard errors. '*' represents significant difference (P < 0.05), and '**' represents extremely significant difference (P < 0.01).

Among the shared genes in the ChIP-seq, RNA-seq, and DAP-seq data, *WRKY28* and *GRP3* were related to SA (**Supplementary Table S11**); *WRKY28* regulates SA biosynthesis by binding to the isochorismate synthase 1 (*ICS1*) promoter (van Verk et al., 2011), and *GRP3* responses to SA (Park et al., 2001). Therefore, we suspect that *MeGI* regulates sex differentiation in flower buds by regulating SA biosynthesis. Previous studies have shown that the biosynthesis of SA mainly involves two pathways (Chen et al., 2009), and *WRKY28* regulated the biosynthesis of SA mainly through effects on the pathway catalyzed by *ICS*, auxin-responsive GH3 family protein PBS3 (*PBS3*), and HXXXD-type acyl-transferase family protein EPS1 (*EPS1*) (**Figure 7A**). Two homologous genes encoding *ICS* (e.g., *ICS1* and *ICS2*) occur in the *Arabidopsis* genome. Based on the transcriptome sequencing results, the mean fragments per kilobase of transcript per million mapped reads (FPKM) values of *ICS1*, *ICS2*, and *PBS3* were significantly lower in the MeGI-OX lines than in the control lines, but no difference in *EPS1* was observed (**Figure 7B**). These results suggest that *WRKY28* inhibits the expression of *ICS1*, *ICS2*, and *PBS3* to reduce SA biosynthesis.

To verify these results in *D. oleifera*, SA content was determined in 7 female and 7 male *D. oleifera* flower buds that were obtained in mid-April. The SA content was significantly higher in male floral buds than in female floral buds (**Figure 7C**), is consistent with repression of biosynthesis of SA by *MeGI* and that lower SA levels promote female development in *D. oleifera.*

## 4 Discussion

Gene transcription is regulated by numerous transcription factors, which are mainly bound to promoters. The identification of transcription factor-binding sites (TFBSs) is important for analyses of their functions (Ferraz et al., 2021). Many methods have been used to study TFBSs, such as ChIP-seq (Zhang et al., 2013; Gu et al., 2019; Iqbal et al., 2020), Cleavage Under Targets and Tagmentation (Kaya-Okur et al., 2019; Tao et al., 2020), DAP-seq (Yang et al., 2019; Cao et al., 2020; Gomez-Cano et al., 2022), EMSAs (Gu et al., 2019; Liu et al., 2019; Li et al., 2020), and yeast one-hybrid assays (Gu et al., 2019; Liu et al., 2019; Li et al., 2020). Among these methods, ChIP-seq is a high-throughput approach to identify TFBSs (Valouev et al., 2008), but it requires high-quality antibodies; the preparation of TF antibodies requires extensive testing, which is time-consuming and laborious (Wang, 2017). The DAP-seq technique has been applied to the identification of DNA-binding sequences using transcription factors expressed *in vitro* (O’Malley et al., 2016). This technology is advantageous in that experiments can be conducted *in vitro* and are not limited by materials or antibodies. However, DAP-seq has the potential for a high false-positive rate. In our study, we used the ChIP-seq and DAP-seq methods to study TFBSs; we combined the results of the two methods to overcome the limitations of each individual method. But in the results of DAP-seq, only a small portion of peaks landed in promoter regions. The possible reasons for this are: (1) DAP-seq is an *in vitro* experiment, and the randomness of protein binding with DNA fragments is large, so there will be less overlap of experimental data; (2) This may be related to the analysis parameters set during the original data analysis, and a large number of binding fragments failed to reach the order of magnitude required for data analysis and were eliminated as non-specific binding fragments.

The pistil and stamen primordia develop in floral buds at the early stage of flower development in most unisexual plants. Subsequently, either the pistil or stamen primordia degenerate at a particular stage because of the complex effects of genetics, physiology (Golenberg and West, 2013), and environmental factors (Adam et al., 2011); eventually, a unisexual flower is formed (Golenberg and West, 2013; Renner, 2014). Researchers have studied the sex determining systems of some typical dioecious plants, such as *Silene latifolia* (Bačovský et al., 2022; Kazama et al., 2022), *Rumex acetosa* (Manzano et al., 2017), and *Asparagus officinalis* (Harkess et al., 2017; Murase et al., 2017; Harkess et al., 2020); they identified many genes related to sex determination and differentiation. Mutations in these genes cause male and female sterility. Coen and Meyerowitz (1991) proposed the ABC model, in which genes control the development of flower organs. Subsequently, Theissen (2001) proposed the ABCDE model, in which A+E, A+B+E, B+C+E, C+E, and D+E type genes regulate the formation of sepals, petals, stamens, carpels, and ovules, respectively (Grimplet et al., 2016). Yang et al. investigated the *MeGI* regulatory network in hexaploid *D. kaki*; they reported that the expression patterns of short vegetative phase (*SVP*) genes are directly controlled by *MeGI*. Concurrently, *SVP* can inhibit the expression of the class B gene *PISTILLATA* (*PI*) during development of the androecium. In the present study, some genes that belonged to the ABCDE model were identified by ChIP-seq, including *APETALA3* (*AP3*) in class B, *AGAMOUS* (*AG*) in class C, *SEPALLATA1/2/3/4* (*SEP1/2/3/4*) in class E, and *SVP*. However, no differences in the expression patterns of these genes were detected in the RNA-seq results. Potential reasons for these lack of differences include slight differences in the *MeGI* sequence among *Diospyros* spp., which may lead to slight functional differences (**Figure 1C**); the high-quality reference genome used in the present study (Sun et al., 2022) was acquired by optimization of a previous Hi-C assembly version (Suo et al., 2020) using BioNano optical mapping data, which are important for the accurate identification of target genes; and different study methods may lead to different results. According to the present findings, although the ABCDE model explains the mechanism of sex regulation in the unisexual flowers of some species, the ABCDE model alone does not fully explain the mechanism of sex regulation in *Diospyros* spp., and the mechanism for regulation of sex differentiation in *Diospyros* spp. requires further study. In addition, both Akagi et al. (2014); Yang et al. (2019) and our study have shown that not all plants with *MeGI* overexpressing vectors exhibit feminized phenotypes, mainly for the following reasons: (1) differences in *MeGI* expression level; (2) incomplete splicing of *MeGI* mRNA resulted in low expression of the functional *MeGI* transcript (Akagi et al., 2014); (3) uncertainty of *MeGI* insertion site.

**Figure 8:**
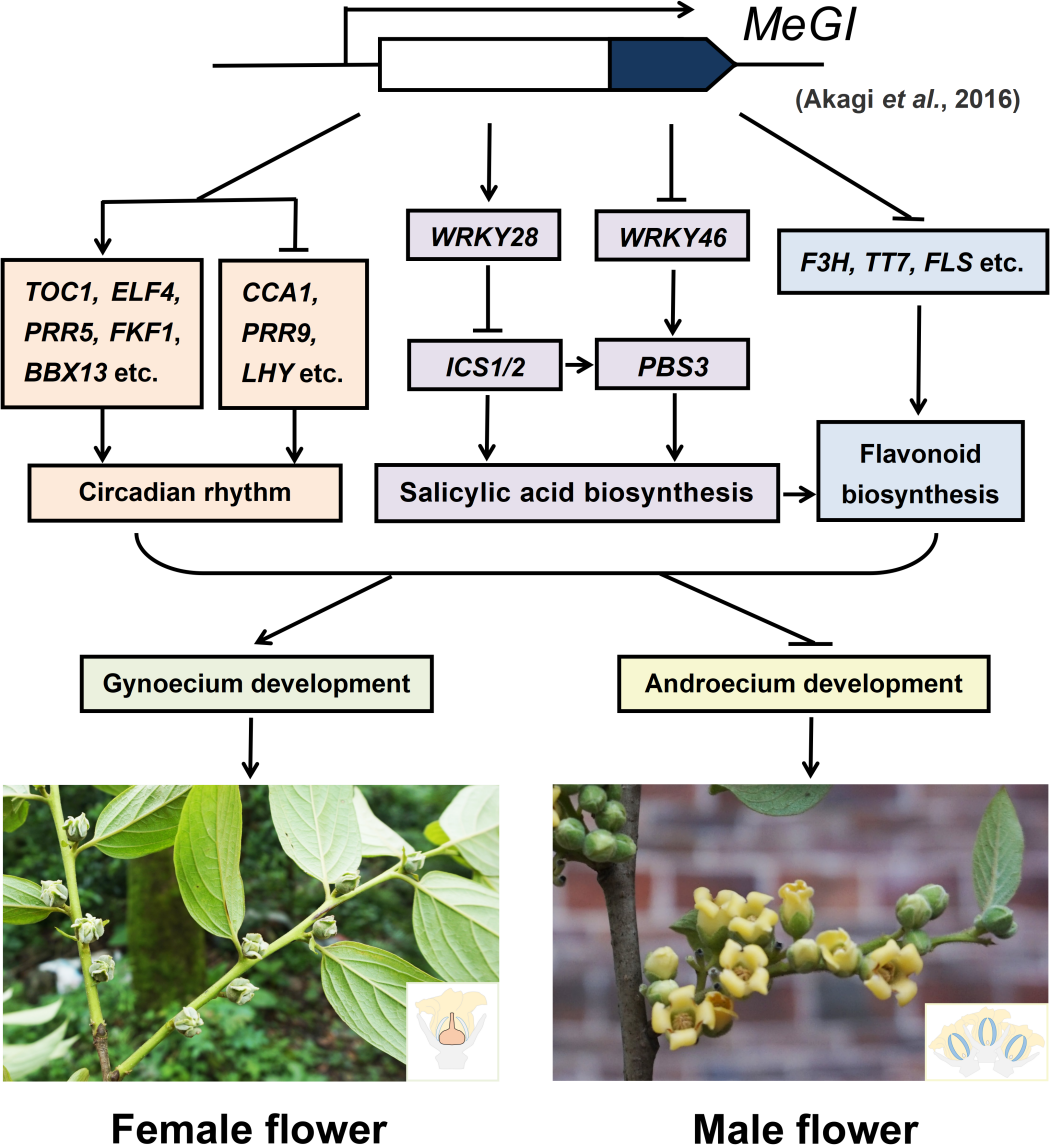
Model of *MeGI*-mediated sex determination in *D. oleifera*.

The sex determination system in plants can be influenced by the external environment, such as light duration, light intensity, and external temperature (Wu et al., 2010). The plant circadian clock is a set of adaptive mechanisms that evolved in response to external factors, such as light and temperature; it has an indispensable role in plant growth and development (Dunlap, 1999). The central oscillator, a core component of the circadian clock, is composed of late elongated hypocotyl (*LHY*), circadian clock associated 1 (*CCA1*), and the family of pseudo-response regulators (*PRRs*), which are important for the maintenance of a stable circadian rhythm (Beckwith and Yanovsky, 2014). Circadian rhythms and the differential expression of related genes reportedly affect sex differentiation in plants. For instance, short day duration inhibits lateral branch differentiation, promotes flower bud differentiation, and induces female flower formation in cucumber (Su, 2020); in andromonoecious *D. kaki*, circadian rhythm-related genes are expressed differently in male and hermaphroditic floral buds at stage 4 (Li et al., 2021). Yang et al. (2019) discovered that *LHY* is nested in the female module by weighted correlation network analysis based on the DEGs of female and male flower buds. In our study, the combined ChIP-seq and RNA-seq results showed that *MeGI* controlled flower bud differentiation partially by regulating circadian rhythm-related genes (e.g., the upregulated genes *TOC1, FKF1, PRR5, ELF4*, and *BBX13*, and the downregulated genes *LHY, PRR9*, and *CCA1*) (**Supplementary Table S12**). The mutations or differential expression of these genes could affect circadian rhythms, thus potentially contributing to sex differentiation in *D. oleifera* flowers (**Figure 8**).

Many studies have shown that plant hormones such as cytokinins (CKs), gibberellin, and ethylene also regulate flower bud sex differentiation; they have played key roles in the evolution of plants from monoecious to dioecious phenotypes (Golenberg and West, 2013). For example, Ming et al. (2020) increased the level of CKs in *Jatropha curcas* L. by transgenic expression of the *Arabidopsis* CK biosynthetic gene (*AtIPT4*); this led to an increase in flower number and the male-to-female ratio. Wang et al. (2022) treated androecious persimmon trees with ethrel and zeatin; they reported that 1,000 mg/L of ethrel and 10 mg/L of zeatin significantly reduced stamen length and pollen grain diameter. Although the effects of plant hormones on sex differentiation of flower buds have been extensively studied, the role of SA in sex differentiation has only been studied in a few species. In rice, the exogenous application of 1,000 µM SA alleviated the decrease in pollen viability and floret fertility induced by high temperature (Zhao et al., 2018). SA is involved in the development of staminodes in *Litsea cubeba* (Lour.) Pers. (Xu et al., 2020). The role of SA was also explored in another study of persimmon. Masuda et al. (2020) showed that SA-associated genes might contribute to the derepression of *OGI* (a male-promoting gene) in hexaploid persimmon, suggesting a male-promoting effect of SA; this was similar to our findings. In our study, *MeGI* directly regulated the expression of *WRKY28* and *GRP3*. *WRKY28* reduced SA synthesis by inhibiting *ICS1/2* expression (van Verk et al., 2011), which further repressed stamen development (**Figure 8**). This is consistent with our findings that the expression levels of *ICS1/2* decreased in *A. thaliana* flowers after overexpression *MeGI*; moreover, the SA content was lower in female floral buds obtained from *D. oleifera* than in corresponding male buds. Additionally, WRKY DNA-binding protein 46 (*WRKY46*) regulates the synthesis of SA by regulating the expression of *PBS3* (van Verk et al., 2011). In our study, *WRKY46* and *PBS3* were concurrently detected in the ChIP-seq and RNA-seq results; both were downregulated in the MeGI-OX lines (**Supplementary Table S10)**. Therefore, we concluded that the decrease in SA level contributed to the *MeGI* function (**Figure 8**).

Furthermore, the flavonoid contents in flower buds were associated with the expression of circadian rhythm-related genes and the SA levels in the buds. For example, *TOC1* (*PRR1*), a gene associated with circadian rhythms, negatively regulates the accumulation of flavonoids in *Carthamus tinctorius* L. (Wu et al., 2022); the application of SA to *Calendula officinalis* L. significantly increases flavonoid content in the inflorescence (Pacheco et al., 2013). In our study, 219 downregulated genes were significantly enriched in the flavonoid biosynthetic pathway (**Supplementary Figure S7B**; **Supplementary Table S13**), and there were no flavonoid biosynthesis-related genes among the 149 upregulated genes. Additionally, most genes in the flavonoid metabolic pathway were downregulated (**Supplementary Figure S5**). Overall, our results were similar to the results of previous studies (**Figure 8**). Furthermore, previous studies have shown that the male and female tissue-specific expression patterns of genes in the flavonoid biosynthetic and metabolic pathways vary among species. Genes associated with flavonoid biosynthesis and metabolism are highly expressed in female *Ginkgo biloba* trees (Liao et al., 2020), whereas the flavonoid biosynthetic pathway in *J. curcas* is associated with the initiation of male flowers (Hui et al., 2017). Nevertheless, high levels of flavonoids appear to induce the development of male floral buds in *D. oleifera.*

In recent years, the persimmon industry has been severely limited by a lack of variety. Because of the high genetic consistency of persimmon in Japan, and the long-term cross-breeding of a few breeding parent combinations, degeneration from inbreeding (e.g., fruit shrinkage and tree weakness) has occurred, which has restricted the development of the persimmon industry (Pei et al., 2015). Yakushiji et al. (1995) observed that certain *D. kaki* plants occasionally produce male flowers from gynoecious structures; similar flower sex changes were found in certain years in our previous observations. These results suggest that the floral sex of *Diospyros* spp. is not absolutely invariable; it may be possible to achieve mutual transformation between male and female flower buds under appropriate conditions, such as exposure to SA *via* spraying or alteration of a plant’s circadian rhythm. These results provide a theoretical basis for artificial induction of superior male persimmon germplasm to efficiently produce high-quality new persimmon cultivars by cross breeding.

## Data availability statement

The datasets presented in this study can be found in online repositories. The names of the repository/repositories and accession number(s) can be found in the article/**Supplementary Material**.

## Author contributions

YM and PS conceived the research; YM, YS, HL, WH, SD, and LW analyzed the experimental data; YM, YW, JY, LY, and YZ validated these experimental data; YM drafted the original manuscript; PS, FL, and JF prepared the revised manuscript. All authors contributed to the article and approved the submitted version.
