# Supplementary material for: Regulatory mechanism of MeGI on sexuality in Diospyros oleifera
Source: Front Plant Sci. 2023 Feb 22;14:1046235. doi: 10.3389/fpls.2023.1046235 (PMC9994623; doi:10.3389/fpls.2023.1046235)
Supplement: Supplementary file 1 [file DataSheet_1.doc]

**Supplementary Table 1.** Probe sequences used in EMSAs.

| Probe | Forward primers (5-3) | Reverse primers (5-3) |
| --- | --- | --- |
| evm.model.Chr11.1131-w | CACAAAAAGAAAAAAAATTA | TAATTTTTTTTCTTTTTGTG |
| evm.model.Chr11.1131-m | CACAACCTTAGGGGGAATTA | TAATTCCCCCTAAGGTTGTG |
| evm.model.Chr5.1036-w | CGAACAAACCAAAATTTAATAATCG | CGATTATTAAATTTTGGTTTGTTCG |
| evm.model.Chr5.1036-m | CGGCAGGAGCGGGGTTTAATAATCG | CGATTATTAAACCCCGCTCCTGCCG |

Supplementary Table 2. Phenotypes of the MeGI-overexpressed and control transgenic lines.

| line | construct | feminization | *MeGI* expression level | mRNA-Seq |
| --- | --- | --- | --- | --- |
| 35S-MeGI-OX-1 | pHB-MeGI | - | + |  |
| 35S-MeGI-OX-2 | pHB-MeGI | - | ++ |  |
| 35S-MeGI-OX-3 | pHB-MeGI | - | + |  |
| 35S-MeGI-OX-4 | pHB-MeGI | - | ++ |  |
| 35S-MeGI-OX-5 | pHB-MeGI | + | ++ | X |
| 35S-MeGI-OX-6 | pHB-MeGI | + | ++ | X |
| 35S-MeGI-OX-7 | pHB-MeGI | - | ++ |  |
| 35S-MeGI-OX-8 | pHB-MeGI | - | ++ |  |
| 35S-MeGI-OX-9 | pHB-MeGI | - | + |  |
| 35S-MeGI-OX-10 | pHB-MeGI | ++ | +++ | X |
| 35S-empty-1 | pHB | - | - | X |
| 35S-empty-2 | pHB | - | - | X |
| 35S-empty-3 | pHB | - | - | X |
| 35S-empty-4 | pHB | - | - |  |
| 35S-empty-5 | pHB | - | - |  |

**Supplementary Table 3.** RNA-seq sequencing and alignment data.

| Sample | Total Clean Reads (M) | Total Mapping(%) | Uniquely Mapping(%) |
| --- | --- | --- | --- |
| Cont_1 | 113.11 | 96.06 | 90.21 |
| Cont_2 | 113.10 | 95.81 | 89.33 |
| Cont_3 | 113.49 | 96.38 | 90.00 |
| MeGI_OX_1 | 117.52 | 95.76 | 88.39 |
| MeGI_OX_2 | 117.53 | 94.72 | 87.46 |
| MeGI_OX_3 | 113.26 | 96.21 | 88.94 |

Supplementary Table 4. Differentially expressed lncRNAs in whole transcriptome analysis.

| Gene ID | log_2_ (MeGI_OX / Cont) | Qvalue | Known/Novel Gene |
| --- | --- | --- | --- |
| AT3G08315 | -1.419983033 | 5.61E-04 | known |
| AT3G54625 | 1.363451774 | 2.60E-04 | known |
| AT5G01215 | 1.296512556 | 1.42E-13 | known |
| AT1G69572 | 1.894315714 | 1.08E-21 | known |
| AT1G75891 | -1.116855569 | 9.48E-03 | known |
| AT2G04852 | 1.074463457 | 2.48E-04 | known |
| BGIG3702_31021 | 1.204847401 | 1.13E-04 | novel |
| BGIG3702_31054 | 1.177766923 | 6.35E-04 | novel |

**Supplementary Table 5.** Differentially expressed miRNAs in whole transcriptome analysis.

| Gene ID | log_2_ (MeGI_OX / Cont) | Qvalue | Known/Novel Gene |
| --- | --- | --- | --- |
| ath-miR158a-3p | 4.975892185 | 0.00532803 | known |
| ath-miR160a-3p | 10.15888013 | 0.023550011 | known |
| ath-miR161.1 | 9.946104651 | 3.62E-06 | known |
| ath-miR170-5p | 9.537108488 | 0.001909996 | known |
| ath-miR171a-5p | 9.537108488 | 0.001909996 | known |
| ath-miR171b-5p | -12.39339046 | 2.03E-07 | known |
| ath-miR172a | -4.778325408 | 5.68E-04 | known |
| ath-miR172b-3p | -4.778325408 | 5.68E-04 | known |
| ath-miR390a-3p | 9.09031273 | 1.33E-34 | known |
| ath-miR390a-5p | -8.925037683 | 1.68E-05 | known |
| ath-miR390b-5p | -8.925037683 | 1.68E-05 | known |
| ath-miR394a | 7.883972772 | 1.13E-04 | known |
| ath-miR394b-5p | 7.883972772 | 1.13E-04 | known |
| ath-miR396b-5p | 11.30937681 | 0 | known |
| ath-miR398b-3p | 10.74465367 | 0.01340259 | known |
| ath-miR398c-3p | 10.74465367 | 0.01340259 | known |
| ath-miR408-3p | -11.44604941 | 6.75E-07 | known |
| ath-miR5012 | 8.835824846 | 0.005406815 | known |
| ath-miR5650 | -11.29920802 | 1.36E-06 | known |
| ath-miR781a | 12.20426546 | 8.12E-07 | known |
| ath-miR781b | 12.20426546 | 8.12E-07 | known |
| ath-miR866-3p | -10.07146236 | 0.028322225 | known |
| novel-ath-miR105-3p | -13.76636622 | 1.88E-06 | novel |
| novel-ath-miR16-3p | 5.332545202 | 9.00E-06 | novel |
| novel-ath-miR2-3p | -8.209362637 | 5.58E-49 | novel |
| novel-ath-miR45-5p | -4.691482824 | 0.048249429 | novel |
| novel-ath-miR63-3p | 1.69421705 | 0.013260552 | novel |
| novel-ath-miR63-5p | 2.448989872 | 0.01340259 | novel |
| novel-ath-miR94-5p | -11.22217627 | 0 | novel |

**Supplementary Table 6.** Differentially expressed miRNAs and their differentially expressed target genes.

| Gene ID | Target Node ID | log_2_ (MeGI_OX / Cont) | Qvalue (MeGI_OX / Cont) | Gene Symbols | Description |
| --- | --- | --- | --- | --- | --- |
| ath-miR390a-5p | AT2G15880 | 1.16 | 1.28E-08 | - | Leucine-rich repeat (LRR) family protein |
|  | AT2G15890 | 1.30 | 9.82E-18 | MEE14 | maternal effect embryo arrest 14 |
| ath-miR390b-5p | AT2G15880 | 1.16 | 1.28E-08 | - | Leucine-rich repeat (LRR) family protein |
|  | AT2G15890 | 1.30 | 9.82E-18 | MEE14 | maternal effect embryo arrest 14 |
| ath-miR158a-3p | AT3G29590 | -2.33 | 0.0276 | AT5MAT | HXXXD-type acyl-transferase family protein |
| novel-ath-miR63-5p | AT5G06510 | -1.68 | 4.21E-26 | NF-YA10 | nuclear factor Y, subunit A10 |
| ath-miR5012 | AT1G51620 | 1.18 | 1.22E-04 | - | Protein kinase superfamily protein |

Supplementary Table 7. Phenotypes of the MeGI-FLAG overexpressed and control transgenic lines.

| line | construct | feminization | *MeGI* expression level | mRNA-Seq |
| --- | --- | --- | --- | --- |
| 1306-MeGI-FLAG-1 | p1306-MeGI-FLAG | + | ++ | X |
| 1306-MeGI-FLAG-2 | p1306-MeGI-FLAG | - | + |  |
| 1306-MeGI-FLAG-3 | p1306-MeGI-FLAG | - | + |  |
| 1306-MeGI-FLAG-4 | p1306-MeGI-FLAG | + | +++ | X |
| 1306-MeGI-FLAG-5 | p1306-MeGI-FLAG | - | + |  |
| 1306-MeGI-FLAG-6 | p1306-MeGI-FLAG | - | ++ |  |
| 1306-MeGI-FLAG-7 | p1306-MeGI-FLAG | - | ++ |  |
| 1306-MeGI-FLAG-8 | p1306-MeGI-FLAG | - | ++ |  |
| 1306-MeGI-FLAG-9 | p1306-MeGI-FLAG | + | +++ | X |
| 1306-MeGI-FLAG-10 | p1306-MeGI-FLAG | - | + |  |
| 1306-MeGI-FLAG-11 | p1306-MeGI-FLAG | - | + |  |
| 1306-MeGI-FLAG-12 | p1306-MeGI-FLAG | + | ++ |  |
| 1306-FLAG-1 | p1306-FLAG | - | - | X |
| 1306-FLAG-2 | p1306-FLAG | - | - | X |
| 1306-FLAG-3 | p1306-FLAG | - | - | X |
| 1306-FLAG-4 | p1306-FLAG | - | - |  |
| 1306-FLAG-5 | p1306-FLAG | - | - |  |

Supplementary Table 8. Genes directly regulated by *MeGI* in DAP-seq analysis.

| Gene ID | avr. FPKM | |  | TAIR annotation | | | |
| --- | --- | --- | --- | --- | --- | --- | --- |
|  | Female | Male |  | ID | Symbols | Description | e-value |
| evm.model.Chr4.1203 | 0.26 | 0.48 |  | - | - | - | - |
| evm.model.Chr7.833 | 9.25 | 7.86 |  | AT2G38360 | PRA1.B4 | prenylated RAB acceptor 1.B4 | 5.74E-106 |
| evm.model.Chr13.746 | 0.04 | 0.00 |  | AT4G17030 | EXLB1 | expansin-like B1 | 1.47E-76 |
| evm.model.Chr9.1141 | 15.27 | 17.60 |  | AT1G27595 | AT1G27595 | symplekin | 0 |
| evm.model.Chr9.1233 | 5.14 | 1.39 |  | AT1G20060 | AT1G20060 | ATP binding microtubule motor family protein | 5.03E-165 |
| evm.model.Chr2.622 | 18.34 | 8.21 |  | AT5G10110 | AT5G10110 | DNA-directed RNA polymerase subunit beta | 7.77E-33 |
| evm.model.Chr5.73 | 0.48 | 0.26 |  | AT4G29360 | AT4G29360 | O-Glycosyl hydrolases family 17 protein | 1.26E-54 |
| evm.model.Chr11.1131 | 25.91 | 3.76 |  | AT2G05520 | GRP3 | glycine-rich protein 3 | 3.07E-6 |
| evm.model.Chr9.402 | 9.25 | 15.75 |  | AT1G17860 | AT1G17860 | Kunitz family trypsin and protease inhibitor protein | 1.38E-62 |
| evm.model.Chr14.1204 | 13.42 | 11.21 |  | AT2G46310 | CRF5 | cytokinin response factor 5 | 1.67E-43 |
| evm.model.Chr2.254 | 0.81 | 1.08 |  | AT3G47180 | AT3G47180 | RING/U-box superfamily protein | 1.81E-20 |
| evm.model.Chr2.437 | 29.87 | 5.15 |  | AT3G18800 | AT3G18800 | transmembrane protein | 2.98E-87 |
| evm.model.Chr11.1378 | 4.91 | 0.98 |  | - | - | - | - |
| evm.model.Chr14.835 | 4.99 | 133.19 |  | AT5G46940 | AT5G46940 | Plant invertase/pectin methylesterase inhibitor superfamily protein | 3.89E-30 |
| evm.model.Chr5.1036 | 19.27 | 10.22 |  | AT4G18170 | WRKY28 | WRKY DNA-binding protein 28 | 1.87E-12 |
| evm.model.Chr14.1262 | 34.73 | 28.98 |  | AT1G01300 | AT1G01300 | Eukaryotic aspartyl protease family protein | 0 |
| evm.model.Chr1.187 | 15.36 | 6.66 |  | AT4G19020 | CMT2 | chromomethylase 2 | 0 |
| evm.model.Chr9.1207 | 0.00 | 0.00 |  | - | - | - | - |
| evm.model.Chr12.1463 | 2.96 | 2.85 |  | AT1G72275 | AT1G72275 | LOW protein: nuclease-like protein | 1.18E-89 |
| evm.model.Chr13.182 | 10.04 | 10.01 |  | AT3G25150 | AT3G25150 | Nuclear transport factor 2 (NTF2) family protein with RNA binding (RRM-RBD-RNP motifs) domain-containing protein | 1.08E-106 |
| evm.model.Chr15.881 | 0.00 | 6.16 |  | AT1G15080 | LPP2 | lipid phosphate phosphatase 2 | 2.45E-128 |
| evm.model.Chr12.1276 | 80.59 | 62.53 |  | AT3G43300 | ATMIN7 | HOPM interactor 7 | 1.8E-27 |
| evm.model.Chr4.2126 | 15.41 | 18.78 |  | AT4G35500 | AT4G35500 | Protein kinase superfamily protein | 0 |
| evm.model.Chr7.62 | 5.11 | 6.27 |  | AT3G58570 | AT3G58570 | P-loop containing nucleoside triphosphate hydrolases superfamily protein | 2.57E-71 |
| evm.model.Chr8.1780 | 147.46 | 117.78 |  | AT3G59540 | AT3G59540 | Ribosomal L38e protein family | 4.92E-42 |
| evm.model.Chr8.137 | 0.00 | 0.00 |  | AT4G35160 | AT4G35160 | O-methyltransferase family protein | 8.31E-21 |
| evm.model.Chr10.862 | 19.25 | 18.00 |  | AT5G25150 | TAF5 | TBP-associated factor 5 | 0 |
| evm.model.Chr10.1039 | 3.12 | 2.89 |  | AT4G11670 | - | DNA topoisomerase 4 subunit B (DUF810) | 4.17E-42 |
| evm.model.Chr15.1539 | 6.52 | 6.97 |  | AT3G54720 | AMP1 | Peptidase M28 family protein | 0 |
| evm.model.Chr4.1229 | 14.28 | 27.41 |  | AT4G30100 | AT4G30100 | P-loop containing nucleoside triphosphate hydrolases superfamily protein | 0 |
| evm.model.Chr15.134 | 8.84 | 5.65 |  | AT5G63060 | AT5G63060 | Sec14p-like phosphatidylinositol transfer family protein | 1.77E-117 |
| evm.model.Chr3.927 | 0.40 | 0.12 |  | AT2G35630 | MOR1 | ARM repeat superfamily protein | 6.12E-26 |
| evm.model.Chr9.645 | 15.42 | 17.18 |  | AT4G17420 | AT4G17420 | Tryptophan RNA-binding attenuator protein-like protein | 1.34E-162 |
| evm.model.Chr13.143 | 196.13 | 85.24 |  | AT3G07390 | AIR12 | auxin-induced in root cultures-like protein | 2.42E-47 |
| evm.model.Chr3.662 | 15.41 | 17.61 |  | AT4G19040 | EDR2 | ENHANCED DISEASE RESISTANCE 2 | 0 |
| evm.model.Chr4.1147 | 8.13 | 3.57 |  | AT2G22490 | CYCD2%3B1 | Cyclin D2%3B1 | 1.23E-83 |
| evm.model.Chr8.1794 | 1.11 | 0.67 |  | - | - | - | - |
| evm.model.Chr1.2727 | 0.00 | 0.00 |  | AT3G52730 | AT3G52730 | ubiquinol-cytochrome C reductase UQCRX/QCR9-like family protein | 8.02E-8 |
| evm.model.Chr15.1003 | 27.42 | 54.17 |  | AT1G80180 | AT1G80180 | hypothetical protein | 1.75E-30 |
| evm.model.Chr10.1124 | 10.19 | 7.80 |  | AT5G52430 | AT5G52430 | hydroxyproline-rich glycoprotein family protein | 6.59E-102 |
| evm.model.Chr4.1801 | 65.84 | 113.66 |  | AT2G02760 | UBC2 | ubiquiting-conjugating enzyme 2 | 4.67E-108 |
| evm.model.Chr7.423 | 2.13 | 2.55 |  | AT3G12250 | TGA6 | TGACG motif-binding factor 6 |  |
| evm.model.Chr3.1742 | 19.01 | 18.70 |  | AT2G45670 | AT2G45670 | calcineurin B subunit-like protein | 0 |
| evm.model.Chr3.1428 | 1.98 | 1.16 |  | AT5G13470 | AT5G13470 | hypothetical protein | 3.09E-19 |
| evm.model.Chr12.1082 | 0.06 | 0.03 |  | AT3G28345 | ABCB15 | ABC transporter family protein | 0 |
| evm.model.Chr12.2191 | 13.97 | 15.83 |  | AT1G31220 | AT1G31220 | Formyl transferase | 5.97E-110 |
| evm.model.Chr12.624 | 10.56 | 12.31 |  | AT5G11010 | AT5G11010 | Pre-mRNA cleavage complex II protein family | 4.77E-168 |
| evm.model.Chr4.392 | 7.95 | 10.20 |  | AT5G19280 | KAPP | kinase associated protein phosphatase | 0 |
| evm.model.Chr12.1086 | 31.81 | 18.73 |  | AT2G19880 | AT2G19880 | Nucleotide-diphospho-sugar transferases superfamily protein | 2.9E-115 |
| evm.model.Chr9.1244 | 45.76 | 50.83 |  | AT5G42150 | AT5G42150 | Glutathione S-transferase family protein | 3.11E-149 |
| evm.model.Chr1.2839.2 | 3.44 | 3.18 |  | AT3G12080 | emb2738 | GTP-binding family protein | 4.71E-79 |
| evm.model.Chr9.1404 | 1.41 | 0.88 |  | - | - | - | - |
| evm.model.Chr11.513 | 0.57 | 0.20 |  | AT4G05420 | DDB1A | damaged DNA binding protein 1A | 1.93E-116 |
| evm.model.Chr5.899 | 11.83 | 12.91 |  | AT5G04510 | PDK1 | 3-phosphoinositide-dependent protein kinase 1 | 8.8E-154 |
| evm.model.Chr7.380 | 254.80 | 179.79 |  | AT5G12250 | TUB6 | beta-6 tubulin | 0 |
| evm.model.Chr14.188 | 15.04 | 13.14 |  | AT3G05940 | AT3G05940 | organic solute transporter ostalpha protein (DUF300) | 1.13E-157 |
| evm.model.Chr11.1103.4 | 19.08 | 32.80 |  | - | - | - | - |
| evm.model.Chr12.1580 | 0.12 | 0.04 |  | - | - | - | - |

**Supplementary Table 9.** Upregulated genes in RNA-seq and genes that occurred in ChIP-seq data.

| Gene ID | Gene Symbol | log_2_ (MeGI_OX / Cont) | Qvalue (MeGI_OX / Cont) |
| --- | --- | --- | --- |
| AT4G37235 | AT4G37235 | 2.937806549 | 0.024911556 |
| AT1G65486 | AT1G65486 | 1.724321666 | 0.001310861 |
| AT3G27809 | AT3G27809 | 1.018353773 | 8.88E-05 |
| AT2G05380 | GRP3S | 2.305344621 | 5.97E-17 |
| AT2G05520 | GRP3 | 1.740178794 | 1.34E-30 |
| AT2G15890 | MEE14 | 1.304603537 | 9.82E-18 |
| AT2G15960 | AT2G15960 | 2.741706517 | 3.08E-23 |
| AT2G18550 | HB21 | 2.00092383 | 1.93E-05 |
| AT2G18700 | TPS11 | 1.199228421 | 8.15E-12 |
| AT2G19850 | AT2G19850 | 2.796469225 | 0.013444274 |
| AT2G20670 | AT2G20670 | 1.04464874 | 1.84E-06 |
| AT2G21660 | GRP7 | 1.978876617 | 7.75E-58 |
| AT2G23770 | LYK4 | 1.187361556 | 0.001461412 |
| AT2G24560 | AT2G24560 | 2.935792093 | 1.10E-14 |
| AT2G30250 | WRKY25 | 1.511835515 | 1.89E-05 |
| AT2G33830 | AT2G33830 | 4.347308051 | 3.64E-191 |
| AT2G36080 | ABS2 | 1.306395978 | 3.41E-12 |
| AT2G38250 | AT2G38250 | 1.414083444 | 0.02981821 |
| AT2G38290 | AMT2 | 1.1321957 | 2.33E-17 |
| AT2G38870 | AT2G38870 | 1.444229931 | 1.93E-04 |
| AT2G39210 | AT2G39210 | 1.12070975 | 1.20E-11 |
| AT2G40080 | ELF4 | 2.3505144 | 1.06E-27 |
| AT2G41090 | AT2G41090 | 1.026102139 | 1.15E-25 |
| AT2G42170 | AT2G42170 | 1.174390083 | 0.00373819 |
| AT2G43510 | TI1 | 2.450933944 | 7.23E-05 |
| AT2G43570 | CHI | 3.426325696 | 2.29E-09 |
| AT2G43590 | AT2G43590 | 2.60316553 | 2.55E-28 |
| AT2G44810 | DAD1 | 2.471464085 | 5.86E-13 |
| AT2G46430 | CNGC3 | 1.490276358 | 1.05E-21 |
| AT3G04000 | AT3G04000 | 1.093159844 | 5.33E-04 |
| AT3G05660 | RLP33 | 2.100745568 | 3.96E-23 |
| AT3G07650 | COL9 | 1.534063212 | 3.10E-38 |
| AT3G10020 | AT3G10020 | 1.252285615 | 1.03E-09 |
| AT3G15630 | AT3G15630 | 1.517583917 | 2.43E-19 |
| AT3G16400 | NSP1 | 1.071032846 | 3.24E-09 |
| AT3G01580 | AT3G01580 | 1.043251821 | 2.15E-04 |
| AT3G01080 | WRKY58 | 3.093291951 | 8.33E-14 |
| AT3G01290 | HIR2 | 2.288096829 | 9.21E-05 |
| AT3G18550 | BRC1 | 1.609561565 | 3.23E-05 |
| AT3G20340 | AT3G20340 | 1.211775239 | 1.42E-06 |
| AT3G22830 | HSFA6B | 1.635186223 | 0.04698238 |
| AT3G26740 | CCL | 1.132041892 | 9.19E-14 |
| AT3G48850 | PHT3;2 | 5.201300642 | 0.007820341 |
| AT3G50260 | CEJ1 | 1.475114501 | 0.040101771 |
| AT3G50470 | HR3 | 2.541555476 | 5.97E-09 |
| AT3G50480 | HR4 | 1.466657534 | 9.40E-08 |
| AT3G51330 | AT3G51330 | 1.06824438 | 1.04E-04 |
| AT3G53460 | CP29 | 1.139348173 | 4.29E-31 |
| AT3G54880 | AT3G54880 | 1.004628385 | 5.00E-12 |
| AT3G55560 | AGF2 | 1.017045965 | 1.15E-05 |
| AT3G55970 | JRG21 | 1.073342918 | 8.29E-05 |
| AT3G57520 | SIP2 | 1.266479349 | 2.49E-34 |
| AT3G59220 | PRN | 1.838754881 | 3.24E-11 |
| AT3G61060 | PP2-A13 | 1.410974457 | 1.15E-12 |
| AT3G62150 | ABCB21 | 1.488917247 | 1.79E-15 |
| AT3G62550 | AT3G62550 | 1.063164855 | 7.71E-07 |
| AT3G63160 | OEP6 | 1.854709191 | 3.17E-55 |
| AT3G63380 | AT3G63380 | 4.682514925 | 8.89E-28 |
| AT4G04330 | RbcX1 | 2.418564182 | 3.72E-17 |
| AT4G18170 | WRKY28 | 3.121706267 | 1.88E-11 |
| AT4G18250 | AT4G18250 | 1.909903255 | 0.044886016 |
| AT4G00140 | EDA34 | 1.294059828 | 0.030894732 |
| AT4G22530 | AT4G22530 | 1.381050155 | 9.01E-11 |
| AT4G23130 | CRK5 | 1.634175748 | 7.30E-06 |
| AT4G23140 | CRK6 | 1.846200101 | 0.001668816 |
| AT4G27950 | CRF4 | 1.040126903 | 3.04E-08 |
| AT4G28490 | HAE | 1.146972411 | 2.56E-15 |
| AT4G32340 | AT4G32340 | 2.514012188 | 4.89E-78 |
| AT4G33960 | AT4G33960 | 1.516270697 | 8.10E-06 |
| AT4G33980 | AT4G33980 | 2.252637377 | 1.62E-20 |
| AT4G36670 | PMT6 | 1.648264353 | 1.40E-07 |
| AT4G36740 | HB40 | 1.825351552 | 0.00492926 |
| AT4G36850 | AT4G36850 | 2.815659244 | 2.31E-34 |
| AT4G37390 | BRU6 | 1.287962192 | 7.75E-14 |
| AT4G38550 | AT4G38550 | 1.01327588 | 1.10E-09 |
| AT5G05600 | AT5G05600 | 1.911774303 | 6.70E-06 |
| AT5G12270 | AT5G12270 | 1.323179201 | 2.49E-22 |
| AT5G14470 | AT5G14470 | 1.020364365 | 0.008940993 |
| AT5G15340 | AT5G15340 | 1.131450485 | 0.002410234 |
| AT5G16010 | AT5G16010 | 1.000007722 | 5.50E-11 |
| AT5G17460 | AT5G17460 | 1.305572712 | 1.03E-04 |
| AT5G01380 | AT5G01380 | 2.653214594 | 1.42E-09 |
| AT5G01210 | AT5G01210 | 1.404941489 | 1.19E-35 |
| AT5G02160 | AT5G02160 | 1.026045707 | 1.01E-13 |
| AT5G18470 | AT5G18470 | 1.791489041 | 4.74E-14 |
| AT5G19120 | AT5G19120 | 1.083497089 | 2.63E-08 |
| AT5G20250 | DIN10 | 1.68939953 | 2.60E-21 |
| AT5G23240 | AT5G23240 | 2.6605928 | 2.13E-45 |
| AT5G24160 | SQE6 | 1.530644714 | 5.99E-04 |
| AT5G24470 | PRR5 | 1.723803073 | 2.02E-35 |
| AT5G24910 | CYP714A1 | 1.927226948 | 2.19E-26 |
| AT5G25810 | tny | 1.124310076 | 0.037927644 |
| AT5G26340 | MSS1 | 1.044828379 | 0.012642712 |
| AT5G37260 | RVE2 | 1.277794081 | 6.19E-25 |
| AT5G42530 | AT5G42530 | 1.650124271 | 1.02E-14 |
| AT5G44210 | ERF9 | 1.008808762 | 8.89E-05 |
| AT5G46330 | FLS2 | 1.388088241 | 1.81E-29 |
| AT5G56550 | OXS3 | 1.081280174 | 2.45E-10 |
| AT5G57630 | CIPK21 | 1.644907302 | 5.28E-11 |
| AT5G61370 | AT5G61370 | 1.182804031 | 6.38E-06 |
| AT5G61380 | TOC1 | 1.198342757 | 3.69E-46 |
| AT5G62360 | AT5G62360 | 1.484362514 | 7.96E-05 |
| AT5G63450 | CYP94B1 | 3.837298202 | 1.54E-16 |
| AT5G64120 | AT5G64120 | 1.205746581 | 1.06E-06 |
| AT5G66790 | AT5G66790 | 1.200516453 | 3.98E-05 |
| AT5G67190 | DEAR2 | 1.15052875 | 4.91E-04 |
| AT1G07430 | HAI2 | 1.161236504 | 4.61E-04 |
| AT1G09970 | LRR XI-23 | 1.025232908 | 1.76E-09 |
| AT1G10340 | AT1G10340 | 1.307625189 | 4.85E-05 |
| AT1G11210 | AT1G11210 | 1.805943413 | 1.41E-16 |
| AT1G11260 | STP1 | 1.61026881 | 9.32E-08 |
| AT1G13110 | CYP71B7 | 1.131868334 | 1.66E-04 |
| AT1G14780 | AT1G14780 | 1.520584593 | 4.27E-10 |
| AT1G14890 | AT1G14890 | 1.118538531 | 3.84E-18 |
| AT1G16850 | AT1G16850 | 1.823856259 | 1.12E-04 |
| AT1G18710 | MYB47 | 1.587626215 | 1.00E-04 |
| AT1G03220 | AT1G03220 | 1.446936094 | 6.41E-15 |
| AT1G03230 | AT1G03230 | 1.074378163 | 3.14E-16 |
| AT1G19540 | AT1G19540 | 1.571034064 | 1.85E-17 |
| AT1G21120 | IGMT2 | 1.968063888 | 0.004454237 |
| AT1G21250 | WAK1 | 1.430122418 | 2.34E-42 |
| AT1G21270 | WAK2 | 1.480616851 | 2.67E-23 |
| AT1G21310 | EXT3 | 1.095633132 | 8.56E-09 |
| AT1G21680 | AT1G21680 | 1.146789889 | 1.20E-15 |
| AT1G22570 | AT1G22570 | 1.33635734 | 2.46E-07 |
| AT1G24625 | ZFP7 | 1.656316759 | 4.78E-05 |
| AT1G25560 | TEM1 | 1.115867857 | 5.94E-11 |
| AT1G26800 | AT1G26800 | 1.036865546 | 0.016417966 |
| AT1G02400 | GA2OX6 | 1.021744463 | 4.96E-04 |
| AT1G28050 | BBX13 | 1.120216299 | 3.01E-15 |
| AT1G48330 | AT1G48330 | 1.283716275 | 3.17E-07 |
| AT1G49500 | AT1G49500 | 3.386628222 | 7.51E-05 |
| AT1G51800 | IOS1 | 1.924175422 | 6.75E-13 |
| AT1G52000 | AT1G52000 | 1.426368876 | 2.52E-30 |
| AT1G53470 | MSL4 | 1.869378914 | 2.60E-06 |
| AT1G55850 | CSLE1 | 1.121101098 | 1.06E-14 |
| AT1G56300 | AT1G56300 | 1.089149933 | 1.44E-21 |
| AT1G58270 | ZW9 | 1.009753251 | 1.62E-06 |
| AT1G58602 | AT1G58602 | 1.480213932 | 2.94E-67 |
| AT1G61810 | BGLU45 | 1.627530294 | 2.46E-04 |
| AT1G64170 | CHX16 | 1.662315885 | 3.88E-09 |
| AT1G66760 | AT1G66760 | 1.719905163 | 5.17E-25 |
| AT1G68050 | FKF1 | 2.712475089 | 7.13E-69 |
| AT1G68500 | AT1G68500 | 1.923209344 | 5.75E-05 |
| AT1G68620 | AT1G68620 | 1.008586942 | 0.042672589 |
| AT1G75490 | AT1G75490 | 1.670589177 | 3.73E-09 |
| AT1G76590 | AT1G76590 | 2.236785783 | 1.65E-18 |
| AT1G76790 | IGMT5 | 3.088494661 | 4.28E-61 |
| AT1G78850 | AT1G78850 | 2.192410807 | 5.86E-21 |

**Supplementary Table 10.** Downregulated genes in RNA-seq and genes that occurred in ChIP-seq data.

| Gene ID | Gene Symbol | log_2_ (MeGI_OX / Cont) | Qvalue (MeGI_OX / Cont) |
| --- | --- | --- | --- |
| AT1G29290 | AT1G29290 | -1.063293648 | 3.38E-04 |
| AT1G32920 | AT1G32920 | -1.203772539 | 0.00178681 |
| AT1G02335 | GL22 | -3.115055492 | 3.14E-35 |
| AT4G15248 | BBX30 | -2.343787689 | 1.37E-05 |
| AT4G36230 | AT4G36230 | -1.736960512 | 1.98E-11 |
| AT2G41415 | AT2G41415 | -1.228477694 | 7.97E-09 |
| AT5G17165 | AT5G17165 | -1.455019156 | 5.41E-11 |
| AT1G48953 | AT1G48953 | -1.305333637 | 0.043882424 |
| AT5G07571 | AT5G07571 | -1.07276726 | 7.92E-05 |
| AT2G01580 | AT2G01580 | -1.622620059 | 0.015642443 |
| AT2G05100 | LHCB2.1 | -1.964245589 | 2.89E-211 |
| AT2G05940 | RIPK | -1.0687342 | 1.65E-13 |
| AT2G20750 | EXPB1 | -1.09085055 | 1.12E-08 |
| AT2G22240 | MIPS2 | -1.846717121 | 2.56E-108 |
| AT2G22470 | AGP2 | -1.24362268 | 2.20E-05 |
| AT2G22970 | SCPL11 | -1.235271866 | 1.17E-21 |
| AT2G23000 | scpl10 | -1.640439586 | 2.74E-23 |
| AT2G23810 | TET8 | -1.300652585 | 1.95E-25 |
| AT2G27080 | AT2G27080 | -1.193364824 | 0.004924158 |
| AT2G27420 | AT2G27420 | -4.877611475 | 2.41E-04 |
| AT2G28110 | FRA8 | -1.429512492 | 1.88E-06 |
| AT2G31750 | UGT74D1 | -1.199525594 | 3.45E-19 |
| AT2G32140 | AT2G32140 | -1.126832294 | 7.69E-09 |
| AT2G32720 | CB5-B | -1.20066351 | 3.49E-26 |
| AT2G35930 | PUB23 | -1.307849039 | 8.13E-10 |
| AT2G37260 | TTG2 | -1.084768069 | 0.012051329 |
| AT2G38470 | WRKY33 | -1.201403378 | 3.54E-04 |
| AT2G39650 | AT2G39650 | -1.021116388 | 2.89E-09 |
| AT2G41380 | AT2G41380 | -1.70081645 | 1.86E-10 |
| AT2G41510 | CKX1 | -1.003765526 | 0.003579205 |
| AT2G42540 | COR15A | -1.104172263 | 6.39E-20 |
| AT2G42990 | AT2G42990 | -1.107871753 | 1.10E-13 |
| AT2G43290 | MSS3 | -1.411813638 | 3.25E-66 |
| AT2G44460 | BGLU28 | -1.146103976 | 3.17E-05 |
| AT2G45040 | AT2G45040 | -1.081604888 | 1.34E-07 |
| AT2G45130 | SPX3 | -1.455782013 | 0.046479525 |
| AT2G46400 | WRKY46 | -1.410603405 | 3.72E-16 |
| AT2G46790 | PRR9 | -1.860766653 | 1.29E-19 |
| AT2G46830 | CCA1 | -1.986316551 | 4.95E-79 |
| AT2G46860 | PPa3 | -1.373065398 | 5.24E-15 |
| AT2G46950 | CYP709B2 | -1.002861878 | 1.15E-07 |
| AT2G47360 | AT2G47360 | -1.505275722 | 1.59E-07 |
| AT3G06890 | AT3G06890 | -1.006430151 | 2.79E-10 |
| AT3G12320 | AT3G12320 | -1.886032302 | 2.80E-50 |
| AT3G13784 | CWINV5 | -2.132168493 | 0.005821933 |
| AT3G14280 | AT3G14280 | -1.048131286 | 0.02131258 |
| AT3G14395 | AT3G14395 | -1.409555093 | 0.004517845 |
| AT3G15760 | AT3G15760 | -1.029700086 | 4.10E-07 |
| AT3G17180 | scpl33 | -2.290885303 | 0.030239842 |
| AT3G17800 | AT3G17800 | -1.108288713 | 1.55E-20 |
| AT3G03470 | CYP89A9 | -1.000087252 | 3.14E-11 |
| AT3G19380 | PUB25 | -1.188049933 | 6.33E-17 |
| AT3G19680 | AT3G19680 | -1.313171741 | 2.30E-21 |
| AT3G21890 | BBX31 | -1.318438329 | 0.025221833 |
| AT3G22370 | AOX1A | -1.004596812 | 4.32E-08 |
| AT3G22840 | ELIP1 | -3.527758029 | 3.89E-29 |
| AT3G26280 | CYP71B4 | -1.203752551 | 0.005474055 |
| AT3G26290 | CYP71B26 | -1.269152569 | 1.12E-17 |
| AT3G27690 | LHCB2.3 | -1.707193554 | 4.88E-54 |
| AT3G44630 | AT3G44630 | -1.106732503 | 8.35E-16 |
| AT3G45640 | MPK3 | -1.31997354 | 1.68E-15 |
| AT3G50060 | MYB77 | -1.16134662 | 2.64E-17 |
| AT3G50560 | AT3G50560 | -1.758153975 | 7.62E-15 |
| AT3G50800 | AT3G50800 | -1.125332125 | 4.04E-05 |
| AT3G50930 | BCS1 | -1.198499549 | 1.99E-15 |
| AT3G51240 | F3H | -1.290583757 | 1.44E-41 |
| AT3G53830 | AT3G53830 | -1.225118917 | 2.04E-13 |
| AT3G55100 | ABCG17 | -1.160287348 | 7.10E-09 |
| AT3G55980 | SZF1 | -1.130474079 | 2.83E-13 |
| AT3G56200 | AT3G56200 | -1.072824281 | 6.10E-20 |
| AT3G57010 | AT3G57010 | -1.051310295 | 2.51E-04 |
| AT3G57020 | AT3G57020 | -1.001445574 | 1.72E-05 |
| AT3G57510 | ADPG1 | -1.510547254 | 2.25E-04 |
| AT3G57530 | CPK32 | -1.015339666 | 7.71E-14 |
| AT3G57540 | AT3G57540 | -2.414371006 | 0.004421782 |
| AT3G57810 | AT3G57810 | -1.228990406 | 1.21E-15 |
| AT3G58060 | AT3G58060 | -1.161247433 | 6.02E-04 |
| AT3G58120 | BZIP61 | -1.15883295 | 1.05E-10 |
| AT3G59060 | PIL6 | -1.396330328 | 1.87E-43 |
| AT3G60550 | CYCP3;2 | -1.45553894 | 1.66E-05 |
| AT3G61220 | SDR1 | -1.198980111 | 6.88E-18 |
| AT3G61910 | NAC066 | -1.427883416 | 0.001143447 |
| AT4G04020 | FIB | -1.312895998 | 1.33E-37 |
| AT4G04750 | AT4G04750 | -1.055215497 | 1.91E-13 |
| AT4G08950 | EXO | -1.486485918 | 1.68E-20 |
| AT4G11280 | ACS6 | -1.214419041 | 3.05E-15 |
| AT4G12730 | FLA2 | -1.063803256 | 4.06E-16 |
| AT4G15530 | PPDK | -1.441431482 | 0.002912867 |
| AT4G16980 | AT4G16980 | -1.308694724 | 2.83E-30 |
| AT4G17490 | ERF6 | -1.051075552 | 0.012134985 |
| AT4G19170 | NCED4 | -1.448128185 | 1.32E-38 |
| AT4G21200 | GA2OX8 | -1.569771901 | 2.53E-07 |
| AT4G01060 | CPL3 | -1.57715495 | 0.023941431 |
| AT4G02410 | AT4G02410 | -1.344514106 | 3.25E-21 |
| AT4G21740 | AT4G21740 | -1.095578907 | 4.62E-14 |
| AT4G22240 | AT4G22240 | -1.206751289 | 1.41E-20 |
| AT4G22880 | LDOX | -1.996611334 | 0.002503557 |
| AT4G24010 | CSLG1 | -1.042451911 | 1.08E-07 |
| AT4G24380 | AT4G24380 | -1.181540288 | 6.69E-22 |
| AT4G25480 | DREB1A | -1.031720089 | 5.04E-05 |
| AT4G27280 | AT4G27280 | -1.095413967 | 3.22E-08 |
| AT4G27654 | AT4G27654 | -1.593714498 | 4.19E-10 |
| AT4G29780 | AT4G29780 | -1.082863589 | 5.75E-13 |
| AT4G30170 | AT4G30170 | -1.448183926 | 9.26E-06 |
| AT4G30270 | XTH24 | -1.955997161 | 9.58E-51 |
| AT4G32105 | AT4G32105 | -1.179247692 | 0.00255848 |
| AT4G34150 | AT4G34150 | -1.226907605 | 2.52E-13 |
| AT4G34320 | AT4G34320 | -1.690469712 | 1.09E-04 |
| AT4G34410 | RRTF1 | -1.360998849 | 9.01E-14 |
| AT4G35060 | HIPP25 | -1.903553451 | 1.57E-05 |
| AT4G35320 | AT4G35320 | -1.203336268 | 4.86E-14 |
| AT4G36640 | AT4G36640 | -1.165168948 | 7.92E-15 |
| AT4G37760 | SQE3 | -1.035771461 | 1.91E-20 |
| AT4G37800 | XTH7 | -1.416518583 | 3.93E-07 |
| AT4G38420 | sks9 | -1.01381088 | 2.32E-07 |
| AT4G39770 | TPPH | -1.503193354 | 0.015015073 |
| AT4G39800 | MIPS1 | -1.724309776 | 9.19E-57 |
| AT5G06510 | NF-YA10 | -1.680060516 | 4.21E-26 |
| AT5G06980 | AT5G06980 | -1.725589565 | 2.32E-18 |
| AT5G07990 | TT7 | -1.623090801 | 1.29E-28 |
| AT5G08640 | FLS1 | -1.268013485 | 4.00E-42 |
| AT5G02580 | AT5G02580 | -1.475535607 | 9.20E-06 |
| AT5G13320 | PBS3 | -1.058666536 | 9.20E-16 |
| AT5G16400 | TRXF2 | -1.03769238 | 2.74E-15 |
| AT5G16570 | GLN1;4 | -1.240252655 | 3.33E-07 |
| AT5G01600 | FER1 | -1.064025684 | 2.93E-14 |
| AT5G02540 | AT5G02540 | -1.447951173 | 2.14E-25 |
| AT5G18270 | ANAC087 | -1.434556187 | 6.99E-05 |
| AT5G21120 | EIL2 | -2.039488618 | 0.017663109 |
| AT5G24030 | SLAH3 | -1.016248549 | 3.58E-10 |
| AT5G24150 | SQP1 | -1.678576528 | 1.85E-43 |
| AT5G37540 | AT5G37540 | -1.048256325 | 3.99E-20 |
| AT5G42380 | CML37 | -1.38215233 | 2.50E-09 |
| AT5G43150 | AT5G43150 | -1.056196245 | 0.001242373 |
| AT5G44400 | AT5G44400 | -1.656912008 | 2.24E-26 |
| AT5G45340 | CYP707A3 | -1.503900151 | 1.51E-15 |
| AT5G45630 | AT5G45630 | -1.030498349 | 1.35E-07 |
| AT5G47230 | ERF5 | -1.16324321 | 0.004340376 |
| AT5G47450 | TIP2;3 | -3.270081215 | 0.020894867 |
| AT5G48880 | KAT5 | -1.019010159 | 5.06E-13 |
| AT5G51990 | CBF4 | -1.153609176 | 2.46E-09 |
| AT5G52750 | AT5G52750 | -1.066120137 | 1.56E-06 |
| AT5G52760 | AT5G52760 | -1.218961586 | 1.44E-05 |
| AT5G55250 | IAMT1 | -1.076053755 | 7.07E-14 |
| AT5G55620 | AT5G55620 | -1.172164265 | 4.00E-05 |
| AT5G57560 | TCH4 | -1.270149274 | 4.26E-05 |
| AT5G57670 | AT5G57670 | -1.50122577 | 5.87E-14 |
| AT5G59130 | AT5G59130 | -1.136121261 | 1.53E-19 |
| AT5G59320 | LTP3 | -1.065814071 | 3.64E-17 |
| AT5G62130 | AT5G62130 | -1.148073184 | 1.85E-43 |
| AT5G62430 | CDF1 | -1.45636749 | 1.35E-04 |
| AT5G62480 | GSTU9 | -3.729710286 | 0.007844725 |
| AT5G64170 | AT5G64170 | -1.587461277 | 4.19E-43 |
| AT5G64770 | RGF9 | -1.241517134 | 0.024703166 |
| AT5G64840 | ABCF5 | -1.068545647 | 5.52E-39 |
| AT5G65690 | PCK2 | -1.084648951 | 8.96E-09 |
| AT5G66675 | AT5G66675 | -1.063680481 | 1.28E-16 |
| AT5G66690 | UGT72E2 | -1.259338297 | 4.12E-19 |
| AT5G66800 | AT5G66800 | -1.127984483 | 9.23E-06 |
| AT5G67030 | ABA1 | -1.455551257 | 6.12E-87 |
| AT5G67140 | AT5G67140 | -1.008107154 | 1.42E-06 |
| AT5G67370 | CGLD27 | -1.372390235 | 4.06E-28 |
| AT1G06000 | AT1G06000 | -1.41294635 | 8.21E-40 |
| AT1G07135 | AT1G07135 | -1.101908253 | 0.012592152 |
| AT1G07180 | NDA1 | -1.524122729 | 7.39E-39 |
| AT1G14520 | MIOX1 | -1.332684746 | 8.74E-19 |
| AT1G18740 | AT1G18740 | -1.08493884 | 5.19E-21 |
| AT1G19190 | AT1G19190 | -2.143418101 | 1.70E-22 |
| AT1G19640 | JMT | -1.022277115 | 5.03E-25 |
| AT1G20500 | AT1G20500 | -2.24649507 | 6.97E-07 |
| AT1G20510 | OPCL1 | -1.027349896 | 4.15E-16 |
| AT1G20823 | AT1G20823 | -1.210530673 | 6.75E-35 |
| AT1G20850 | XCP2 | -1.260732633 | 2.41E-15 |
| AT1G21460 | SWEET1 | -1.06123238 | 9.15E-23 |
| AT1G23205 | AT1G23205 | -1.144296657 | 1.24E-12 |
| AT1G26790 | AT1G26790 | -1.822298096 | 0.001459449 |
| AT1G01250 | AT1G01250 | -1.20714422 | 4.97E-08 |
| AT1G01520 | ASG4 | -1.37607954 | 5.93E-04 |
| AT1G01060 | LHY | -2.288506481 | 3.43E-66 |
| AT1G02900 | RALF1 | -1.252523491 | 9.21E-09 |
| AT1G01580 | FRO2 | -1.505027072 | 1.37E-06 |
| AT1G04670 | AT1G04670 | -1.212670644 | 4.50E-05 |
| AT1G01070 | UMAMIT28 | -1.042742176 | 9.71E-05 |
| AT1G02390 | GPAT2 | -1.138587405 | 1.26E-06 |
| AT1G04250 | AXR3 | -1.419962007 | 1.31E-07 |
| AT1G04240 | SHY2 | -1.200293952 | 9.26E-05 |
| AT1G28370 | ERF11 | -1.305491011 | 3.47E-17 |
| AT1G28570 | AT1G28570 | -1.140770916 | 4.13E-14 |
| AT1G29440 | SAUR63 | -1.216388794 | 0.001429133 |
| AT1G30020 | AT1G30020 | -1.252352792 | 9.37E-05 |
| AT1G30350 | AT1G30350 | -2.428003535 | 1.08E-50 |
| AT1G49640 | AT1G49640 | -1.529803703 | 0.001430971 |
| AT1G51700 | DOF1 | -1.327206089 | 4.48E-29 |
| AT1G52565 | AT1G52565 | -1.029106199 | 1.44E-04 |
| AT1G57980 | AT1G57980 | -1.016673851 | 0.001311987 |
| AT1G58420 | AT1G58420 | -1.222158675 | 2.63E-15 |
| AT1G60190 | PUB19 | -1.847811494 | 3.15E-36 |
| AT1G61667 | AT1G61667 | -1.271330476 | 0.004152274 |
| AT1G61890 | AT1G61890 | -1.041714871 | 7.47E-17 |
| AT1G62710 | BETA-VPE | -2.156332567 | 2.12E-38 |
| AT1G65870 | AT1G65870 | -1.755608896 | 5.26E-05 |
| AT1G66390 | MYB90 | -1.567335451 | 0.047293394 |
| AT1G67360 | AT1G67360 | -1.126731863 | 9.51E-19 |
| AT1G67990 | TSM1 | -1.383345198 | 3.01E-25 |
| AT1G69500 | CYP704B1 | -1.362943914 | 2.48E-21 |
| AT1G72220 | AT1G72220 | -1.423966485 | 1.82E-05 |
| AT1G72240 | AT1G72240 | -1.138236822 | 1.55E-04 |
| AT1G72430 | AT1G72430 | -1.00677729 | 4.36E-06 |
| AT1G73370 | SUS6 | -1.093532313 | 8.75E-14 |
| AT1G73540 | NUDT21 | -1.170454152 | 2.92E-17 |
| AT1G73550 | AT1G73550 | -1.274225799 | 5.19E-05 |
| AT1G73600 | AT1G73600 | -1.510062819 | 1.69E-38 |
| AT1G76600 | AT1G76600 | -1.058383788 | 4.30E-10 |
| AT1G76620 | AT1G76620 | -1.134430583 | 5.20E-10 |
| AT1G78290 | SNRK2-8 | -1.193628207 | 1.49E-04 |
| AT1G78570 | RHM1 | -1.229336869 | 1.06E-36 |
| AT1G79270 | ECT8 | -1.058789396 | 4.92E-10 |
| AT1G80130 | AT1G80130 | -1.161044189 | 2.52E-07 |
| AT1G80840 | WRKY40 | -1.001109727 | 6.44E-09 |

**Supplementary Table 11.** GO functional annotation of *GRP3* and *WRKY28.*

| Gene ID (Gene symbol) | GO Molecular Function | GO Cellular Component | GO Biological Process |
| --- | --- | --- | --- |
| AT2G05520 (*GRP3*) | [GO:0005515](http://amigo.geneontology.org/amigo/term/GO:0005515#/report/gene-detail/F20FTSCCKF3553_ARAjtyN/plant/gene/nosts/_blank)   protein binding | [GO:0005576](http://amigo.geneontology.org/amigo/term/GO:0005576#/report/gene-detail/F20FTSCCKF3553_ARAjtyN/plant/gene/nosts/_blank)   extracellular region | [GO:0008361](http://amigo.geneontology.org/amigo/term/GO:0008361#/report/gene-detail/F20FTSCCKF3553_ARAjtyN/plant/gene/nosts/_blank)   regulation of cell size  [GO:0009269](http://amigo.geneontology.org/amigo/term/GO:0009269#/report/gene-detail/F20FTSCCKF3553_ARAjtyN/plant/gene/nosts/_blank)   response to desiccation  [GO:0009723](http://amigo.geneontology.org/amigo/term/GO:0009723#/report/gene-detail/F20FTSCCKF3553_ARAjtyN/plant/gene/nosts/_blank)   response to ethylene  [GO:0009737](http://amigo.geneontology.org/amigo/term/GO:0009737#/report/gene-detail/F20FTSCCKF3553_ARAjtyN/plant/gene/nosts/_blank)   response to abscisic acid  [GO:0009751](http://amigo.geneontology.org/amigo/term/GO:0009751#/report/gene-detail/F20FTSCCKF3553_ARAjtyN/plant/gene/nosts/_blank)   response to salicylic acid  [GO:0009826](http://amigo.geneontology.org/amigo/term/GO:0009826#/report/gene-detail/F20FTSCCKF3553_ARAjtyN/plant/gene/nosts/_blank)   unidimensional cell growth  [GO:0010044](http://amigo.geneontology.org/amigo/term/GO:0010044#/report/gene-detail/F20FTSCCKF3553_ARAjtyN/plant/gene/nosts/_blank)   response to aluminum ion  [GO:0048364](http://amigo.geneontology.org/amigo/term/GO:0048364#/report/gene-detail/F20FTSCCKF3553_ARAjtyN/plant/gene/nosts/_blank)   root development |
| AT4G1817 (*WRKY28*) | [GO:0003677](http://amigo.geneontology.org/amigo/term/GO:0003677#/report/gene-detail/F20FTSCCKF3553_ARAjtyN/plant/gene/nosts/_blank)   DNA binding  [GO:0003700](http://amigo.geneontology.org/amigo/term/GO:0003700#/report/gene-detail/F20FTSCCKF3553_ARAjtyN/plant/gene/nosts/_blank)   DNA-binding transcription factor activity  [GO:0043565](http://amigo.geneontology.org/amigo/term/GO:0043565#/report/gene-detail/F20FTSCCKF3553_ARAjtyN/plant/gene/nosts/_blank)   sequence-specific DNA binding | [GO:0005634](http://amigo.geneontology.org/amigo/term/GO:0005634#/report/gene-detail/F20FTSCCKF3553_ARAjtyN/plant/gene/nosts/_blank)   nucleus | [GO:0006355](http://amigo.geneontology.org/amigo/term/GO:0006355#/report/gene-detail/F20FTSCCKF3553_ARAjtyN/plant/gene/nosts/_blank)   regulation of transcription, DNA-templated  [GO:0042659](http://amigo.geneontology.org/amigo/term/GO:0042659#/report/gene-detail/F20FTSCCKF3553_ARAjtyN/plant/gene/nosts/_blank)   regulation of cell fate specification |

**Supplementary Table 12.** Genes associated with circadian rhythms in the combined ChIP-seq and RNA-seq analyses.

| Gene ID | Gene Symbol | log_2_ (MeGI_OX / Cont) | Qvalue (MeGI_OX / Cont) | Description |
| --- | --- | --- | --- | --- |
| AT2G46790 | PRR9 | -1.861 | 1.29E-19 | pseudo-response regulator 9 |
| AT2G46830 | CCA1 | -1.986 | 4.95E-79 | circadian clock associated 1 |
| AT5G24470 | PRR5 | 1.724 | 2.02E-35 | two-component response regulator-like protein |
|  |  |  |  |
| AT5G61380 | TOC1 | 1.198 | 3.69E-46 | CCT motif -containing response regulator protein |
|  |  |  |  |
| AT5G62430 | CDF1 | -1.456 | 1.35E-04 | cycling DOF factor 1 |
| AT1G26790 | AT1G26790 | -1.822 | 0.001459449 | Dof-type zinc finger DNA-binding family protein |
|  |  |  |  |
| AT1G01060 | LHY | -2.289 | 3.43E-66 | Homeodomain-like superfamily protein |
|  |  |  |  |
| AT1G28050 | BBX13 | 1.120 | 3.01E-15 | B-box type zinc finger protein with CCT domain-containing protein |
|  |  |  |  |
| AT1G68050 | FKF1 | 2.712 | 7.13E-69 | flavin-binding, kelch repeat, f box 1 |
|  |  |  |  |

**Supplementary Table 13.** Genes associated with the flavonoid biosynthetic pathway in the combined ChIP-seq and RNA-seq analyses.

| Gene ID | Gene Symbol | log_2_ (MeGI_OX / Cont) | Qvalue (MeGI_OX / Cont) | Description |
| --- | --- | --- | --- | --- |
| AT3G51240 | F3H | -1.290583757 | 1.44E-41 | flavanone 3-hydroxylase |
| AT4G22880 | LDOX | -1.996611334 | 0.002503557 | leucoanthocyanidin dioxygenase |
|  |  |  |  |
| AT5G07990 | TT7 | -1.623090801 | 1.29E-28 | Cytochrome P450 superfamily protein |
|  |  |  |  |
| AT5G08640 | FLS1 | -1.268013485 | 4.00E-42 | flavonol synthase 1 |
