# Supplementary material for: Regulatory mechanism of MeGI on sexuality in Diospyros oleifera
Source: Front Plant Sci. 2023 Feb 22;14:1046235. doi: 10.3389/fpls.2023.1046235 (PMC9994623; doi:10.3389/fpls.2023.1046235)
Supplement: Supplementary file 2 [file DataSheet_2.doc]

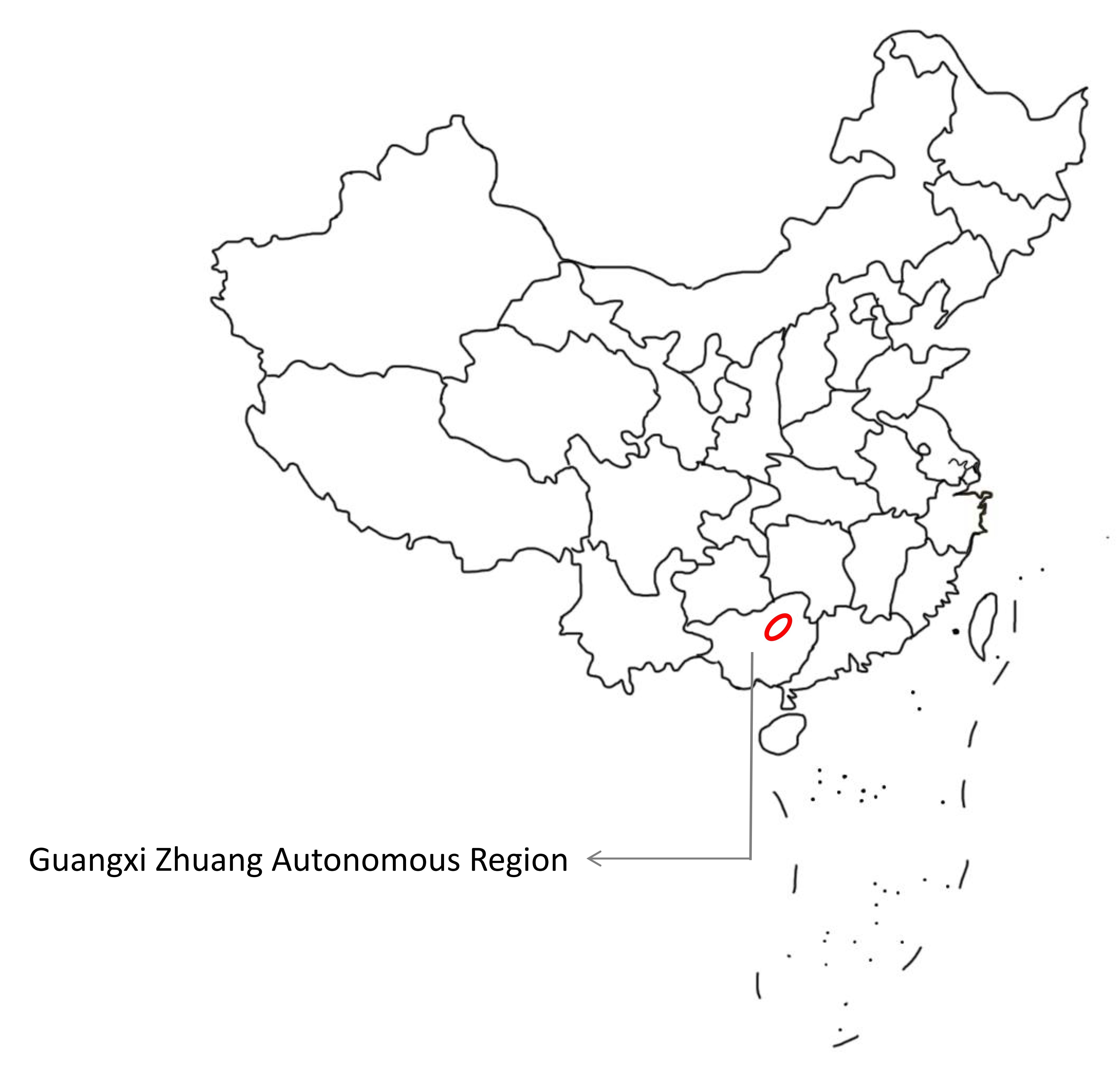


**Supplementary Figure 1.** Sampling site of *D. oleifera* flower buds.


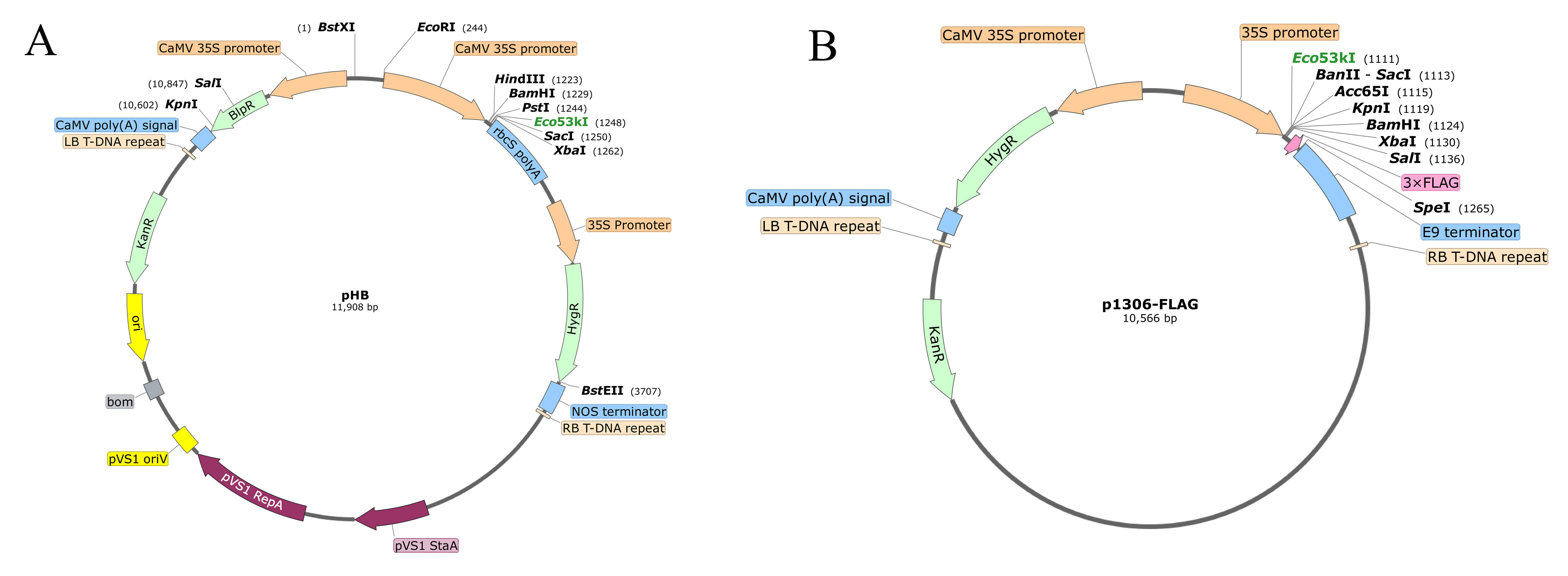


**Supplementary Figure 2.** Plasmid structure of genetic transformation in *Arabidopsis thaliana*. (A) pHB and (B) p1306-FLAG.


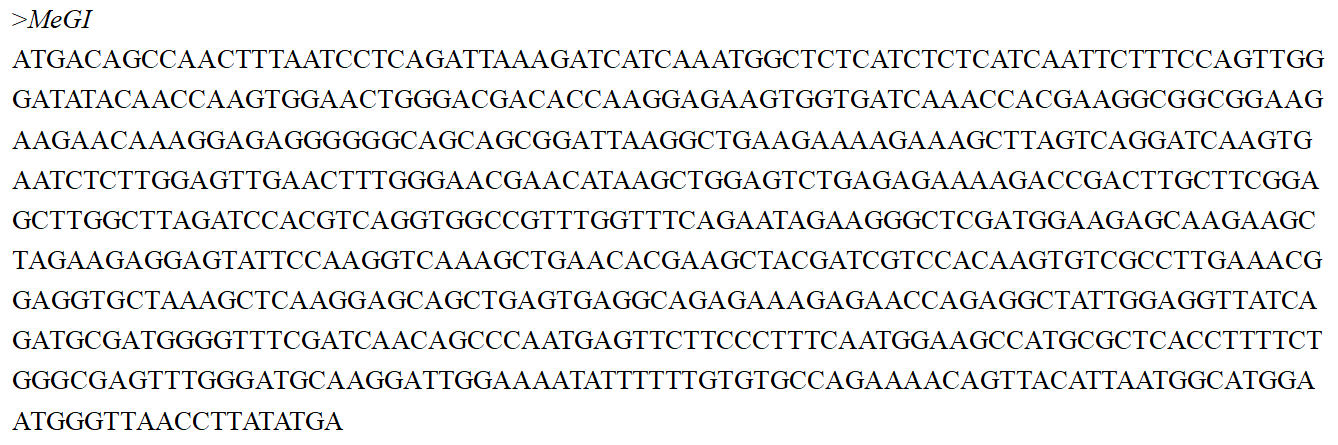


**Supplementary Figure 3.** *MeGI* Open reading frame sequence in *D. oleifera*.


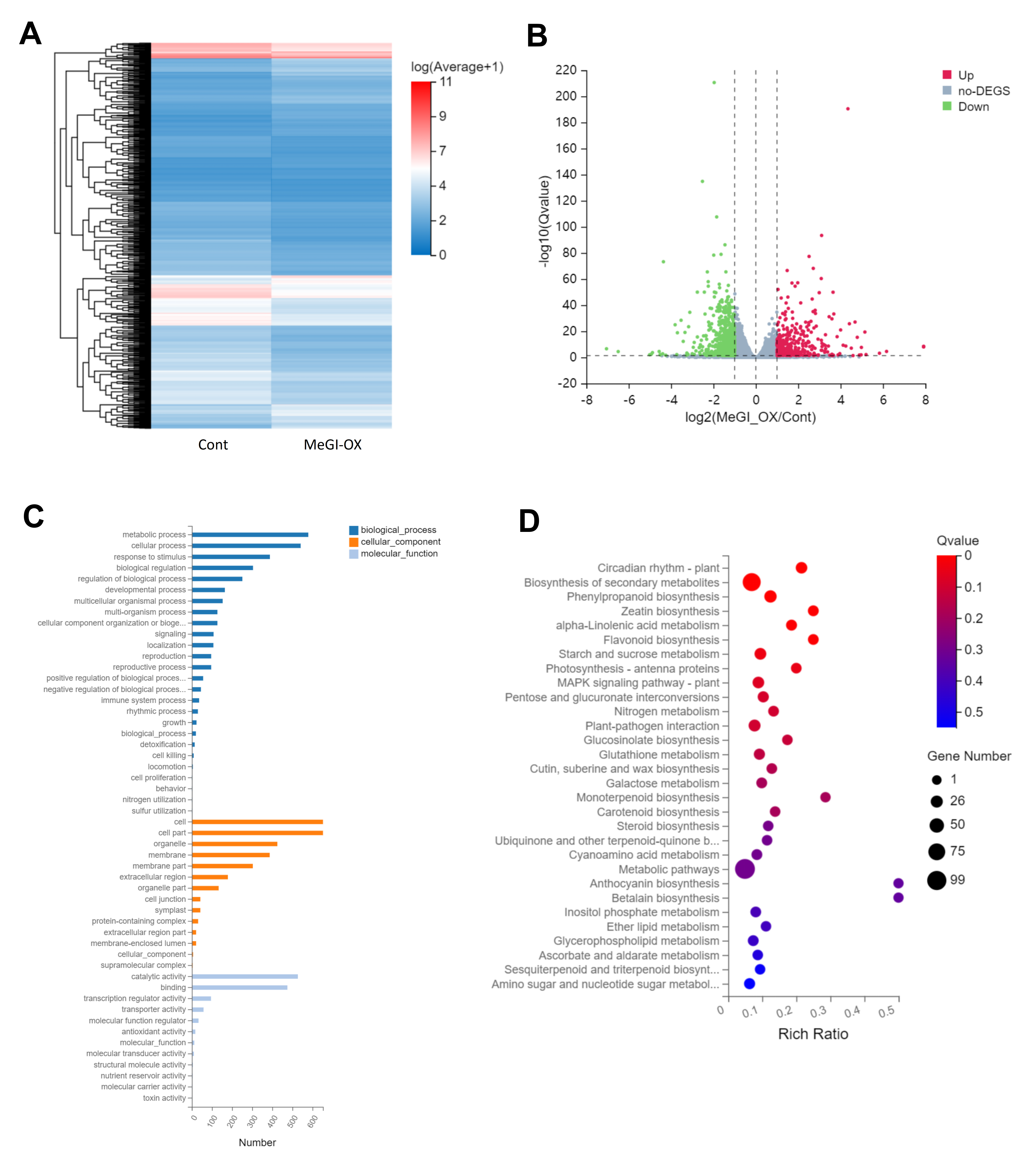


**Supplementary Figure 4.** RNA-seq analysis. (A) Clustering of genes based on the average FPKM of control and MeGI-OX lines. (B) Distribution of differentially expressed genes based on the Qvalue of control and MeGI-OX lines. (C) GO classification analysis of differentially expressed genes. (D) KEGG enrichment analysis of differentially expressed genes.


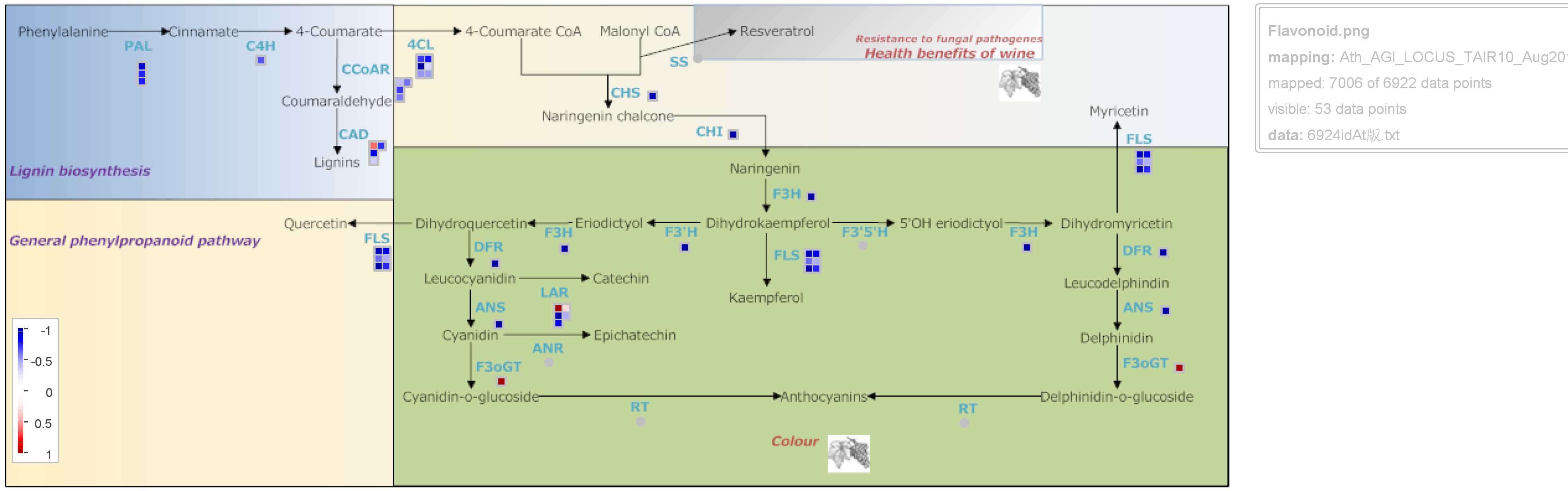


**Supplementary Figure 5.** Flavonoid metabolism and differential expression profiles in pHB-MeGI and pHB-empty lines (blue, pHB-empty biased; red, pHB-MeGI biased). Each square represents a differentially expressed gene (DEG). For more information on MAPMAN diagram interpretation, see Usadel et al. (2009).


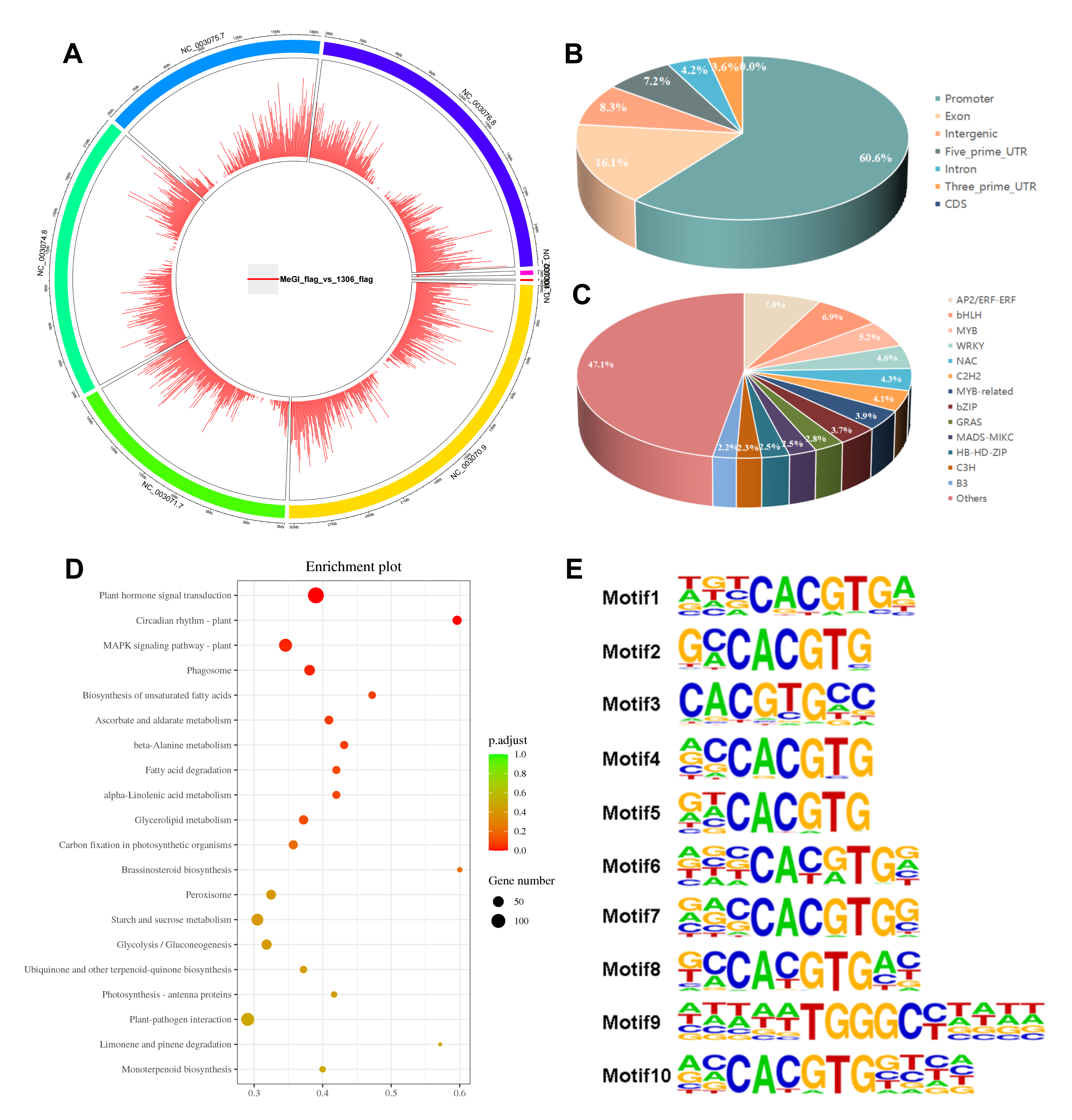


**Supplementary Figure 6.** *MeGI* ChIP-seq analysis. (A) Distribution of MeGI-binding sites along chromosomes of *D. oleifera*. (B) Distribution of MeGI-binding sites in genic and intergenic regions. (C) Transcription factor prediction of MeGI-regulated target genes. (D) KEGG enrichment analysis of MeGI-regulated target genes. (E) Nucleotide motifs recognized by *MeGI* in ChIP-seq.
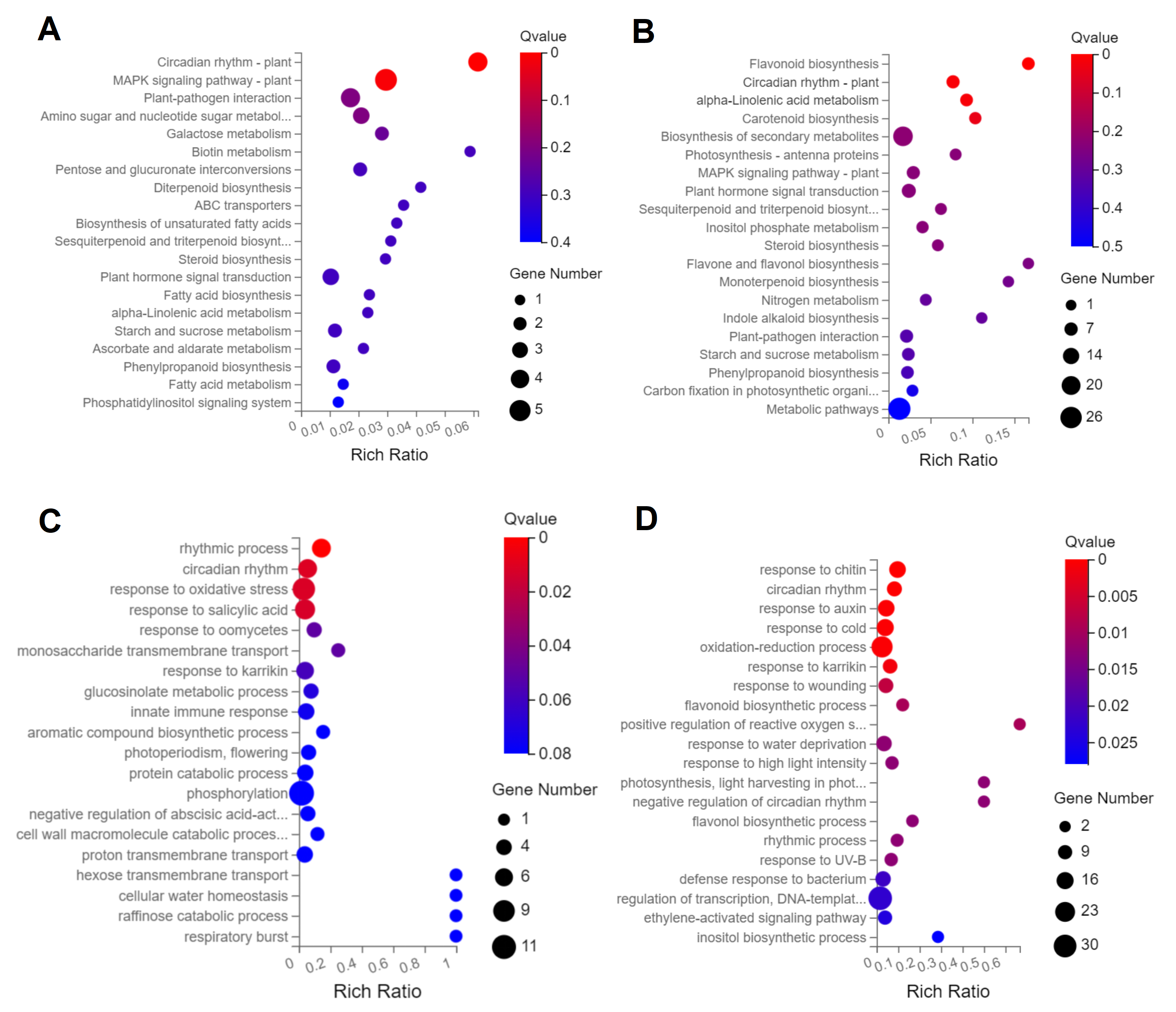


**Supplementary Figure 7.** KEGG and GO enrichment analyses of genes concurrently identified in ChIP-seq and RNA-seq. (A and B) KEGG enrichment analysis of the up-regulated (A) and down-regulated (B) genes. (C and D) GO enrichment analysis of the up-regulated (C) and down-regulated (D) genes.


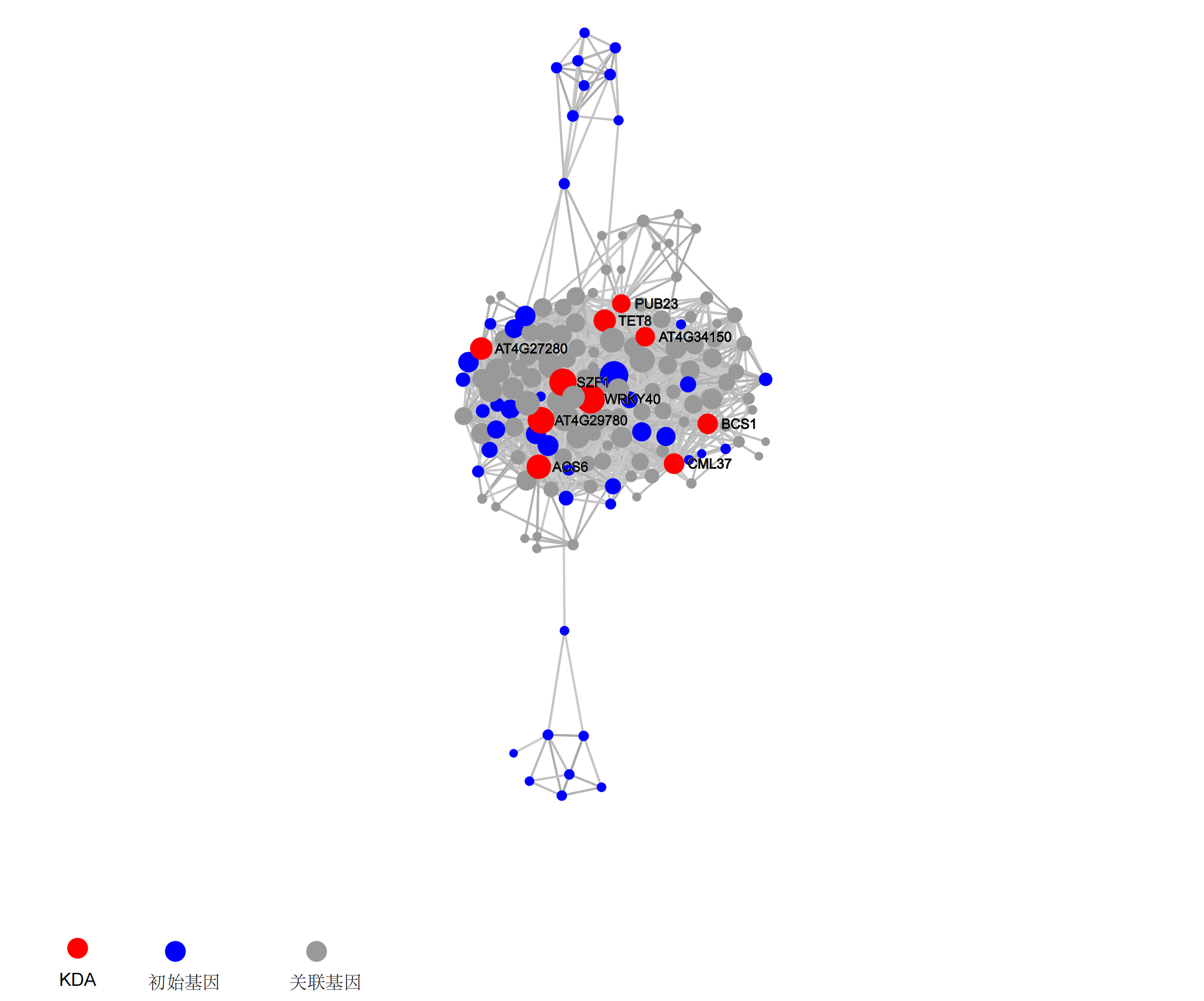


**Supplementary Figure 8.** Key driver analysis of 219 downregulated genes.
